# FGFR inhibition augments anti–PD-1 efficacy in murine FGFR3-mutant bladder cancer by abrogating immunosuppression

**DOI:** 10.1172/JCI169241

**Published:** 2024-01-16

**Authors:** Atsushi Okato, Takanobu Utsumi, Michela Ranieri, Xingnan Zheng, Mi Zhou, Luiza D. Pereira, Ting Chen, Yuki Kita, Di Wu, Hyesun Hyun, Hyojin Lee, Andrew S. Gdowski, John D. Raupp, Sean Clark-Garvey, Ujjawal Manocha, Alison Chafitz, Fiona Sherman, Janaye Stephens, Tracy L. Rose, Matthew I. Milowsky, Sara E. Wobker, Jonathan S. Serody, Jeffrey S. Damrauer, Kwok-Kin Wong, William Y. Kim

**Affiliations:** 1 Lineberger Comprehensive Cancer Center, University of North Carolina, Chapel Hill, North Carolina, USA.; 2 Perlmutter Cancer Center, New York University, New York, New York, USA.; 3 Department of Internal Medicine, College of Medicine, Chungnam National University, Daejeon, South Korea.; 4 Department of Medicine,; 5 Department of Pathology and Laboratory Medicine,; 6 Department of Microbiology and Immunology,; 7 Department of Genetics, and; 8 Department of Pharmacology, University of North Carolina at Chapel Hill, Chapel Hill, North Carolina, USA.

**Keywords:** Oncology, Cancer immunotherapy, Mouse models

## Abstract

The combination of targeted therapy with immune checkpoint inhibition (ICI) is an area of intense interest. We studied the interaction of fibroblast growth factor receptor (FGFR) inhibition with ICI in urothelial carcinoma (UC) of the bladder, in which *FGFR3* is altered in 50% of cases. Using an FGFR3-driven, Trp53-mutant genetically engineered murine model (*UPFL*), we demonstrate that *UPFL* tumors recapitulate the histology and molecular subtype of their *FGFR3*-altered human counterparts. Additionally, *UPFL1* allografts exhibit hyperprogression to ICI associated with an expansion of T regulatory cells (Tregs). Erdafitinib blocked Treg proliferation in vitro, while in vivo ICI-induced Treg expansion was fully abrogated by FGFR inhibition. Combined erdafitinib and ICI resulted in high therapeutic efficacy. In aggregate, our work establishes that, in mice, co-alteration of *FGFR3* and *Trp53* results in high-grade, non–muscle-invasive UC and presents a previously underappreciated role for FGFR inhibition in blocking ICI-induced Treg expansion.

## Abstract 2

FGFR inhibition cooperates with PD-1 checkpoint blockade to reverse anti-PD-1 induced Treg expansion and block progression of murine FGFR3-mutant bladder tumors.

## Graphical abstract

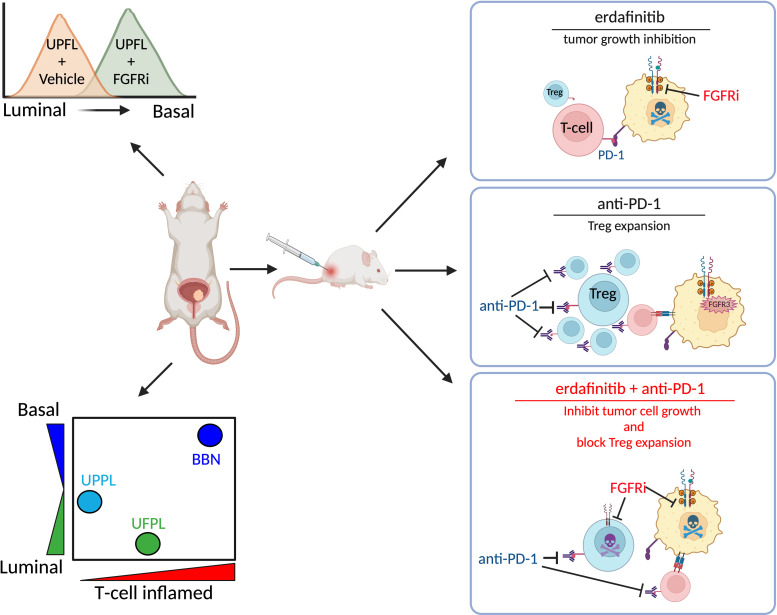

## Introduction

Bladder cancer is the sixth most common cancer in the United States, with an estimated 82,290 new cases and an estimated 16,710 deaths in 2023 (1). At diagnosis, approximately 75% of bladder cancers are non–muscle-invasive (NMIBC), while 25% are de novo muscle-invasive (MIBC) (2). The initial treatment for NMIBC is transurethral resection of bladder tumor that can be followed by treatment with intravesical therapy with Bacillus Calmette-Guerin (BCG) in patients deemed to be at high risk for recurrence. Additionally, the FDA recently approved pembrolizumab for patients who are unresponsive or intolerant to BCG. Other alternatives include cystectomy or treatment with intravesical chemotherapy.

The genetics and transcriptomics of bladder cancer have now been well studied. Upwards of 70% of low-grade NMIBC and 15% of MIBC have genetic alterations in the fibroblast growth factor receptor 3 (*FGFR3*) gene, including activating point mutations or gene fusions (3–6). *FGFR3* alterations predominantly activate the MAPK pathway to promote cell proliferation and survival via ligand-independent dimerization. In bladder cancer, *FGFR3^S249C^* is the predominant hotspot mutation, potentially related to APOBEC-induced mutagenesis (7, 8). The transcriptomic subtypes of MIBC also show a bias in *FGFR3* alteration frequency, with the luminal/luminal papillary (LumP) subtype being enriched for *FGFR3* alterations in multiple subtype classification systems (9–15), while *FGFR3* alterations are enriched in the UROMOL class 1 and class 3 NMIBC subtypes (16). While bladder tumors with a luminal subtype and/or *FGFR3* mutations have both been associated with a non–T cell–inflamed tumor microenvironment, whether *FGFR3* alterations functionally mediate this non–T cell–inflamed phenotype has not been directly explored (17, 18).

Previous works have examined the role of *FGFR3* mutations in murine models of bladder cancer and, with the exception of a recent report (19), have consistently demonstrated that *FGFR3* mutations alone are not sufficient to promote urothelial tumorigenesis. However, *FGFR3* mutations are permissive for the development of carcinoma in situ or high-grade tumors when combined with SV40 large T antigen, *PTEN* loss, or the carcinogen *N*-butyl-*N*-(4-hydroxybutyl)nitrosamine (BBN) (19–22). In the context of BBN, transgenic mice overexpressing *FGFR^K644E^* had decreased neutrophil infiltration, but no other immune cell phenotypes or tumor microenvironment characterization was performed (22).

The FGFR inhibitor erdafitinib is FDA approved for patients with FGFR-altered, advanced urothelial carcinoma (UC) that has progressed during or following prior platinum-containing chemotherapy (23). Despite the relatively non–T cell–inflamed tumor microenvironment, our group and Wang et al. have shown that patients with advanced UC with or without *FGFR3* alterations respond equally well to immune checkpoint inhibition (ICI), perhaps due to a lower level of immunosuppression by stromal elements (24, 25). Functionally, pharmacologic FGFR inhibition or *FGFR3* knockdown in cell culture leads to an increased expression of IFN pathway genes, suggesting that FGFR3 signaling suppresses proinflammatory cytokine secretion in a cell-autonomous manner (24, 26). Nonetheless, to our knowledge, no prior work has examined how *FGFR3* alterations functionally affect the tumor microenvironment nor how acute FGFR inhibition may cooperate with ICI in *FGFR3*-altered bladder cancer.

In this work, we report a mouse model (*UPFL*) that demonstrates cooperativity between the activating hotspot *FGFR^S249C^* mutation and the *Trp53^R270H^* mutation, to reliably produce high-grade NMIBC when expressed in uroplakin 3a–expressing (Upk3a-expressing) cells. Recapitulating human disease, *UPFL* tumors are papillary in histology and transcriptionally similar to both UROMOL class 1 and consensus LumP molecular subtypes (11, 16). Exploration of FGFR3-driven immunobiology demonstrated that *UPFL* tumors have an intermediate T cell–inflamed immune contexture relative to our previously reported BBN (basal) and *UPPL* (luminal) models. We derived a syngenic cell line (*UPFL1*) that allows for transplantable tumor studies to test the interaction between FGFR inhibition and ICI via PD-1 inhibition. *UPFL1* syngeneic tumors were sensitive to erdafitinib and interestingly demonstrated hyperprogression with single-agent anti–PD-1 treatment. In contrast, the combination of erdafitinib and anti–PD-1 worked significantly better than either alone. Flow cytometry demonstrated that while anti–PD-1 treatment of *UPFL1* tumors increased the number of regulatory T cells (Tregs), perhaps accounting for the hyperprogression seen in that model, combined treatment with erdafitinib and anti–PD-1 fully abrogated this Treg increase, suggesting that FGFR inhibition may be able to reverse anti–PD-1–induced immunosuppression. Moreover, erdafitinib treatment was sufficient to block Treg proliferation in vitro. In aggregate, our work establishes that dual *FGFR3* and *Trp53* alteration initiates high-grade, non–muscle-invasive, autochthonous murine bladder tumors with an intermediate T cell–inflamed phenotype and that erdafitinib cooperates with PD-1 checkpoint blockade to reverse anti–PD-1–induced Treg expansion and to block progression.

## Results

### UPFL mice develop papillary, high-grade NMIBCs.

**Figure 1:**
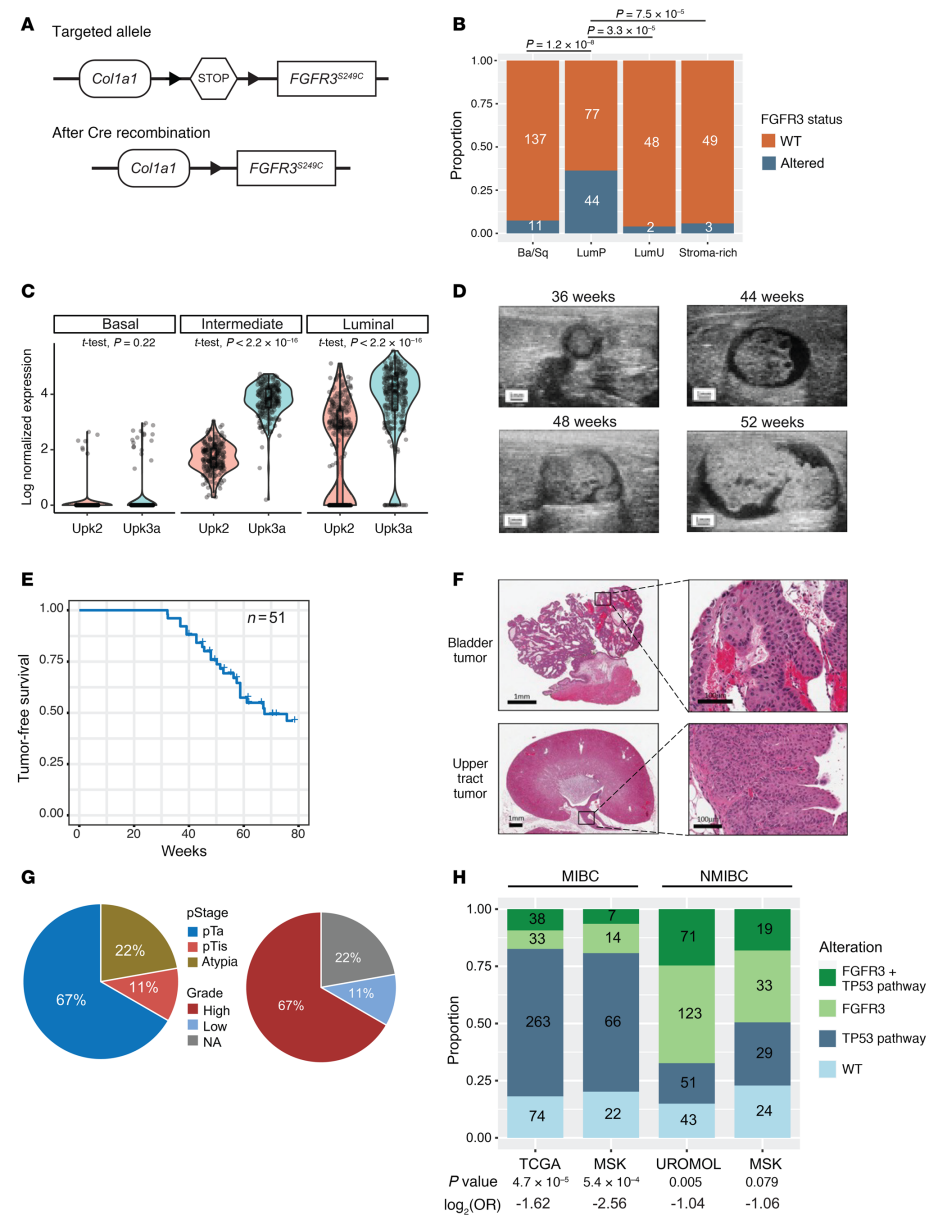
*UPFL* mice develop papillary, high-grade, non–muscle-invasive bladder cancers. (**A**) Schematic of the targeted and recombined *Col1a1-LSL-FGFR3^S249C^* locus. (**B**) Proportion of TCGA-BLCA project tumors within each consensus molecular subtype with FGFR3 mutations. Inset numbers annotate actual patient numbers. (**C**) Violin plots of log_2_-normalized *Upk2* and *Upk3a* RNA expression in basal, intermediate, and luminal cells of mouse urothelium. Two-sided *t* test–derived *P* values were calculated between *Upk2* and *Upk3a* within each cell type. Tumor-free survival and tumor growth were monitored by ultrasound imaging. (**D**) Representative serial ultrasound images of the bladder in a *UPFL* mouse, demonstrating bladder tumor growth (*n* = 51). (**E**) Kaplan-Meier curve of tumor-free survival of 51 total mice. (**F**) Photomicrographs of a bladder tumor and an upper tract urothelial carcinoma (UTUC) of the proximal ureter. Scale bars: 1 mm (left) and 100 μm (right). (**G**) Percentage of tumors of indicated pathologic T stage and grade (*n* = 9). (**H**) Stacked bar plots representing the co-occurrence of *FGFR3* and p53 pathway alterations (*TP53*, *ATM*, *RB1*, *MDM2*, *E2F3*, *ATR*) within MIBC (TCGA and MSK [2014]) and NMIBC (UROMOL and MSK [2017]). Two-sided Fisher’s exact test was performed to calculate significance and log_2_-transformed odds ratios were generated to determine association, with log_2_(OR) < 0 indicating mutual exclusivity.

To understand the role of FGFR3 in bladder cancer biology, we knocked in the cDNA of human *FGFR3* encoding the S249C mutation under control of a LoxP-Stop-LoxP cassette into the collagen type 1, α1 (*Col1a1*) locus to generate mice harboring the *Col1a1*-LSL-*FGFR3^S249^* allele (hereafter called *LSL-FGFR3^S249C^*) (Figure 1A). Prior studies examining the effect of mutant *FGFR3* in bladder cancer have routinely constitutively expressed the gene transgenically under control of the *Upk2* promoter (19, 20, 22). Transgenic overexpression carries the caveats of inappropriate temporal expression (i.e., during development). We wished to examine the effect of *FGFR3^S249C^* activation in adult mice, in a spatiotemporally relevant manner. Based on the evidence that *FGFR3* alterations are more frequently seen in the luminal molecular subtypes (Figure 1B) and single-cell RNA-seq (scRNA-seq) data showing that *Upk3a* is significantly more highly expressed than *Upk2* in luminal/umbrella and intermediate urothelial cells of the normal mouse urothelium (Figure 1C), we drove Cre recombinase expression from the *Upk3a* promoter in a tamoxifen-inducible manner. Additionally, with the exception of a single recent study (19), prior studies have shown that FGFR3 activation alone in the urothelium is not sufficient for tumorigenesis (20–22); therefore, we crossed the *LSL-FGFR3^S249C^* mice with *LSL-Trp53^R270H^* mice (27), *Upk3a*-CreERT2 mice, and LSL-Luc mice (28) to generate *Upk3a-Cre^ERT2^; Trp53^LSL-R270H/+^; LSL-FGFR3^S249C/+^; Rosa26^LSL-Luc/+^* mice (hereafter called *UPFL*). At 8 to 10 weeks of age, *UPFL* mice were administered tamoxifen to activate Cre in the urothelium by oral gavage and monitored for bladder tumor formation via ultrasound (Figure 1D). During the observation window, 47% of *UPFL* mice developed tumors, with a median time to tumor formation of 49 weeks (Figure 1E). Gross inspection of the bladder tumors revealed tumors to be papillary (Supplemental Figure 1A; supplemental material available online with this article; https://doi.org/10.1172/JCI169241DS1), which was verified upon histologic examination (Figure 1F and Supplemental Figure 1, B–E). We also found evidence of upper tract UC (UTUC) (Figure 1F) in 22% of mice, consistent with a known enrichment of FGFR3 alterations in UTUC (29). Histologically, 67% of bladder tumors assessed were high grade and 67% were Ta, with 11% being Tis only (Figure 1G).

While *FGFR3* and *TP53* alterations are mutually exclusive in MIBC (The Cancer Genome Atlas [TCGA] data set, co-altered frequency = 3.7%, 2-sided Fisher’s exact test *P* < 0.001), they are not consistently mutually exclusive in NMIBC. Indeed, when *TP53* is assessed as a pathway, *TP53* pathway members are co-mutated with *FGFR3* relatively frequently. In the UROMOL NMIBC data set, we found that 24.6% (71/288) of samples were co-altered for both *FGFR3* and one of the publication-defined *TP53* pathway genes, while *FGFR3* and the *TP53* pathway were altered alone in 51 samples (17.7%) and 123 samples (42.7%), respectively. Similarly, when evaluating an NMIBC cohort from Memorial Sloan Kettering (MSK), *FGFR3* is co-altered with p53 pathway alterations in 19 out of 105 of tumors (18%), while remaining mutually exclusive in MIBC (Figure 1H) (16, 30–32).

### UPFL tumors are associated with luminal gene expression patterns.

**Figure 2:**
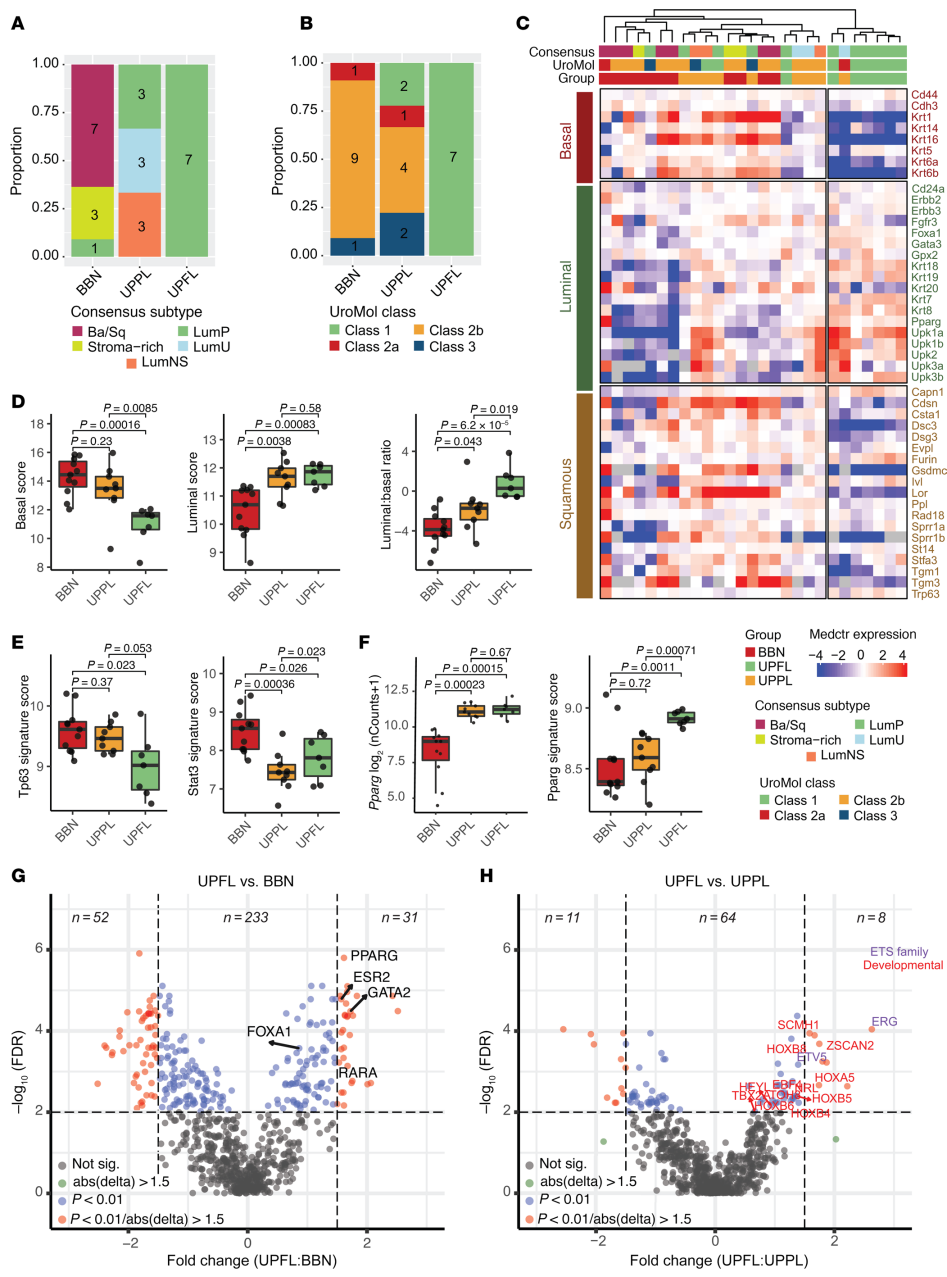
UPFL tumors are associated with luminal expression patterns. (**A**) Proportion of BBN, UPPL, and UPFL primary tumors of the indicated RNA consensus MIBC molecular subtype. (**B**) Proportion of BBN, UPPL, and UPFL primary tumors of the indicated RNA UROMOL NMIBC molecular subtype. (**C**) Heatmap of unsupervised clustering of BBN, UPPL, and UPFL primary tumors by canonical basal and luminal genes. (**D**) Box-and-whisker plots of normalized expression of basal and luminal score as well as luminal to basal score ratio of BBN, UPPL, and UPFL primary tumors. (**E**) Box-and-whisker plots of normalized expression of *Krt6a* and *Upk1a* of BBN, UPPL, and UPFL primary tumors. (**F**) Box-and-whisker plots of *PPARG* and PPARG gene signature (17, 25) of BBN, UPPL, and UPFL primary tumors. All box-and-whisker plots show the IQR and midline at the median. Error bars represent Q1/Q3 ± (1.5 × IQR). Two-sided *t* tests followed by Bonferroni’s correction to account for multiple comparisons were performed; the *P* values are shown above the given comparison. (**G**) Volcano plot of regulon activity between UPFL versus BBN primary tumors. (**H**) Volcano plot of regulon activity between UPFL versus UPPL primary tumors. The *x* axis represents the log_2_(fold change) between UPFL and BBN (**G**) or UPPL (**H**) and the *y* axis is the Benjamini-Hochberg false discovery rate for the given gene.

It has now been repeatedly demonstrated that bladder cancer is a heterogeneous disease with multiple molecular subtypes. While subtyping schema differ, there is a broad consensus around the features defining intrinsic luminal and basal-like subtypes of muscle-invasive disease (9–15). Prior studies have also consistently documented an enrichment of *FGFR3* alterations in the MIBC luminal (specifically LumP) molecular subtype (9, 10, 14, 15), and the NMIBC UROMOL class 1 and class 3 subtypes (16). Following transcriptome profiling by bulk RNA-seq, the *UPFL* expression data were merged with data on BBN and *UPPL* tumors from Saito et al. (33) to characterize its similarity to both MIBC and NMIBC molecular subtypes. Consensus subtype calling was performed on the merged cohort of primary murine bladder tumor models (*UPFL*, BBN, and *UPPL*) and we found that 100% of *UPFL* tumors were most correlated to the consensus LumP subtype. Of the remaining tumors, the majority of BBN tumors were basal/squamous (Ba/Sq), and *UPPL* tumors were distributed across LumP, luminal unstable (LumU), and luminal nonspecified (LumNS) (Figure 2A). Because the consensus subtypes were developed for MIBC and the *UPFL* tumors are NMIBC, we also examined the molecular subtypes using the NMIBC UROMOL classifier (16). The UROMOL classifier assigned all *UPFL* tumors to UROMOL class 1, with a majority of BBN tumors identified as UROMOL class 2b, and again the *UPPL* tumors were heterogeneously dispersed, with representation in all 4 UROMOL classes (Figure 2B). Lindskrog et al. reported that *FGFR3* alterations were enriched in both the UROMOL class 1 and class 3 subtypes, with class 1 tumors more likely to be Ta and having a longer interval to recurrence (Supplemental Figure 2A) (16). Together, these results demonstrate that FGFR3 activation can drive RNA expression patterns that reflect UROMOL class 1 and consensus LumP bladder tumor subtypes, both of which have relatively good prognosis.

We next co-clustered the *UPFL* tumors with the BBN and *UPPL* primary tumors (33) using a canonical list of luminal and basal-like genes and saw that the BBN and *UPPL* tumors had distinct patterns of gene expression, as we have previously described (33). The majority of *UPFL* tumors, however, clustered alone with expression patterns that appeared equally luminal but less basal than our previously published luminal *UPPL* model (Figure 2C). Quantification of luminal and basal scores derived from Choi et al. (9) demonstrated that *UPFL* tumors had a similar luminal score, but a significantly lower average basal score, which in turn resulted in a higher luminal score–to–basal score ratio than *UPPL* tumors (Figure 2D), demonstrating that constitutively active FGFR3 signaling promotes luminal gene expression but also suppresses basal gene expression. Examination of canonical basal transcriptional signatures (p63, STAT3) (9) showed *UPFL* tumors had suppressed basal transcription signaling compared with BBN (Figure 2E). While *Pparg* expression itself was similarly elevated in *UPPL* and *UPFL* tumors, a multigene PPARG transcriptional signature (17, 34) demonstrated that *UPFL* tumors had a significantly more activated PPARG pathway relative to BBN and *UPPL* (Figure 2F). The finding with *Pparg* suggested we look at broader transcriptional networks rather than just individual genes.

To this end, we performed regulon (35) analysis comparing *UPFL* to both BBN and *UPPL* tumors. We saw a large number of differential regulons between *UPFL* and BBN tumors, many of which reflected established differences between basal and luminal tumors (i.e., FOXA1, PPARG, ESR2) (Figure 2G). In contrast, *UPFL* and *UPPL* tumors had very few differential regulons. However, we were struck by Erg being the most differentially upregulated regulon in *UPFL* versus *UPPL* tumors and that another of the ETS transcription factor family, ETV5, was also highly upregulated in *UPFL* tumors (relative to *UPPL* tumors) (Figure 2H). Additionally, a number of developmentally related regulons (HOX genes/TBX2) were upregulated in *UPFL* tumors relative to *UPPL*. Two HOX gene families were represented in the differently expressed regulons and included HOXA (UPFL vs. BBN) and HOXB (UPFL vs. BBN and UPFL vs. UPPL) regulons. These observations are in keeping with the Höglund group’s report of high expression of HOXA and HOXB genes in the Lum UrobasalA subtype, which is enriched for FGFR3-mutant tumors (36).

### scRNA-seq of erdafitinib-treated UPFL tumors confirms that oncogenic FGFR3 drives luminal gene expression across the spectrum of basal to luminal tumor cells.

**Figure 3:**
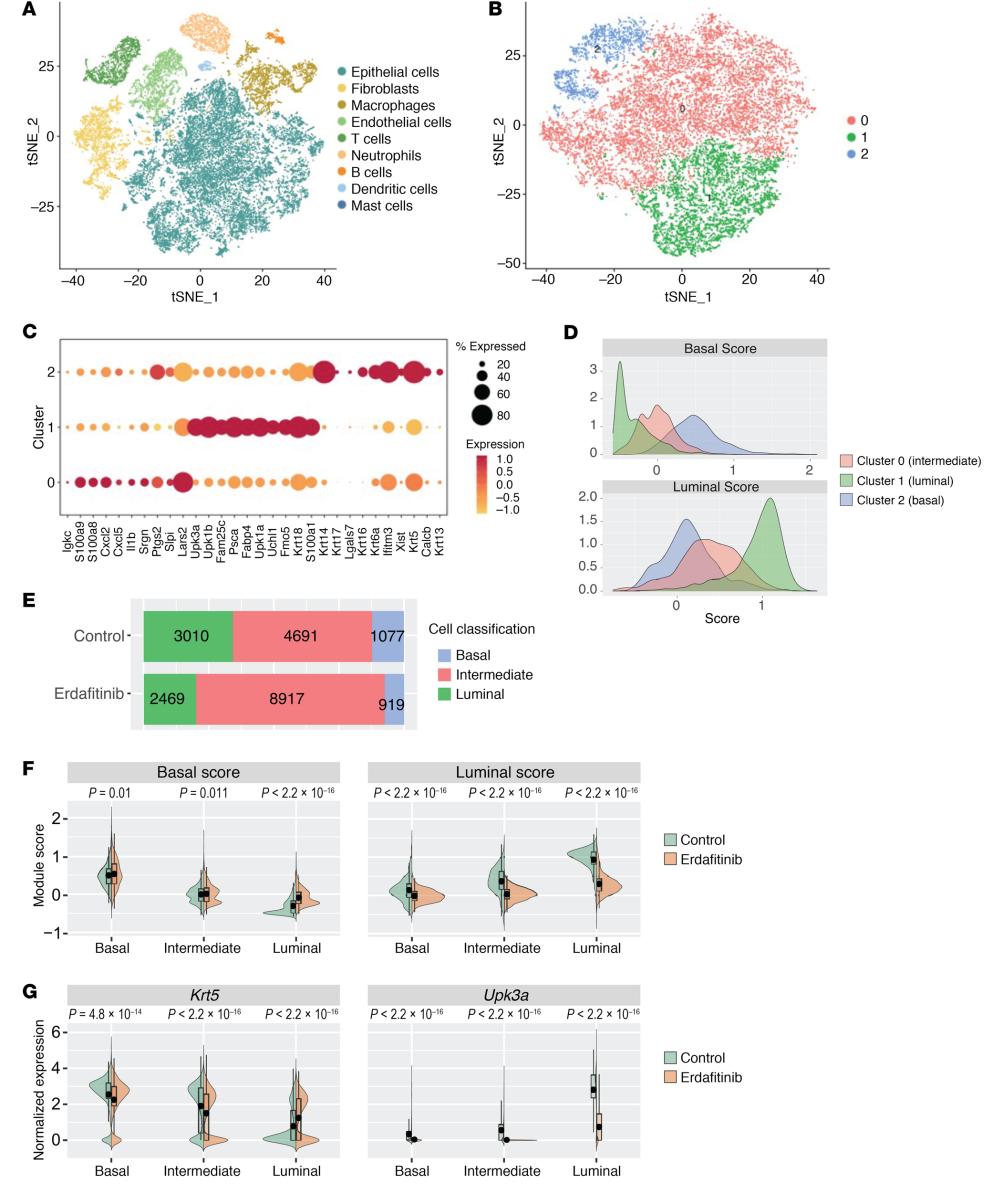
FGFR3 promotes a luminal expression pattern and suppresses the basal transcriptional program across all urothelial cell types, but most prominently in luminal cells. (**A**) tSNE plot of scRNA-seq data of combined control (*n* = 2) and erdafitinib-treated (*n* = 2) tumors. (**B**) tSNE plot of scRNA-seq data of epithelial cells of combined control (*n* = 2) and erdafitinib-treated (*n* = 2) tumors clustered on basal and luminal genes, demonstrating 3 clusters. (**C**) Percentage and level of RNA expression of differentially expressed genes across the indicated clusters. (**D**) Histograms of basal and luminal scores of the indicated clusters from **B**. (**E**) Proportion of epithelial cell type (basal, intermediate, luminal) of control or erdafitinib-treated tumors demonstrates expansion of intermediate cells in erdafitinib-treated tumors. (**F**) Back-to-back violin plots of basal and luminal scores by cell type (basal, intermediate, luminal). (**G**) Back-to-back violin plots of *Krt5* and *Upk3a* RNA expression by cell type (basal, intermediate, luminal). Two-sided *t* tests were performed, with the *P* values shown above the given comparison.

Our findings and work from others support the notion that FGFR3 activation promotes a luminal phenotype. However, whether this finding from bulk RNA-seq is due to an expansion of luminal cells or whether *FGFR3* mutations drive a luminal expression pattern across all tumor cells remains unresolved. In order to more directly assess the role oncogenic FGFR3 was playing in subtype-specific cells, we treated tumor-bearing *UPFL* mice with vehicle or erdafitinib, a small-molecule inhibitor of FGFR (*n* = 2 per group). Following 1 week of treatment, we harvested and dissociated the tumors for scRNA-seq using the 10× Genomics platform. A majority of the isolated cells, as expected, were computationally designated epithelial (Figure 3A). We next separately clustered the epithelial cells using basal and luminal gene markers (9) and identified 3 epithelial groups (Figure 3B). Assessment of the most differentially expressed genes for each epithelial cluster allowed us to assign clusters 0, 1, and 2 to intermediate, luminal, and basal urothelial cells, respectively (Figure 3C). In alignment with the cell identities, calculation of the basal and luminal scores for each cluster demonstrated that cluster 2 had the highest basal gene expression score, while cluster 1 had the highest luminal score and cluster 0 had an intermediate basal and luminal score (Figure 3D).

We next examined the effect of erdafitinib on basal, intermediate, and luminal cell proportion. Erdafitinib-treated tumors appeared to have a larger proportion of intermediate cells at the expense of both luminal and basal cell types (Figure 3E). To better understand how erdafitinib was influencing the basal and luminal transcriptional programs within each epithelial cell type, we calculated the basal and luminal scores for the erdafitinib-treated cells and compared them to their matched vehicle-treated counterpart. While erdafitinib treatment upregulated the basal score, its most pronounced effect was the suppression of the luminal score, specifically within luminal cells (Figure 3F). We saw a similar pattern of gene expression change when specifically looking at erdafitinib’s effect on *Krt5* and *Upk3a* expression across luminal, intermediate, and basal cell types, confirming the basal and luminal score findings (Figure 3G). Taken together, these data demonstrate that FGFR3 promotes a luminal expression pattern and suppresses the basal transcriptional program across all urothelial cell types, but most prominently in luminal cells.

### UPFL tumors are enriched for cytokine signaling relative to UPPL tumors and have an intermediate T cell–inflamed immune contexture.

**Figure 4:**
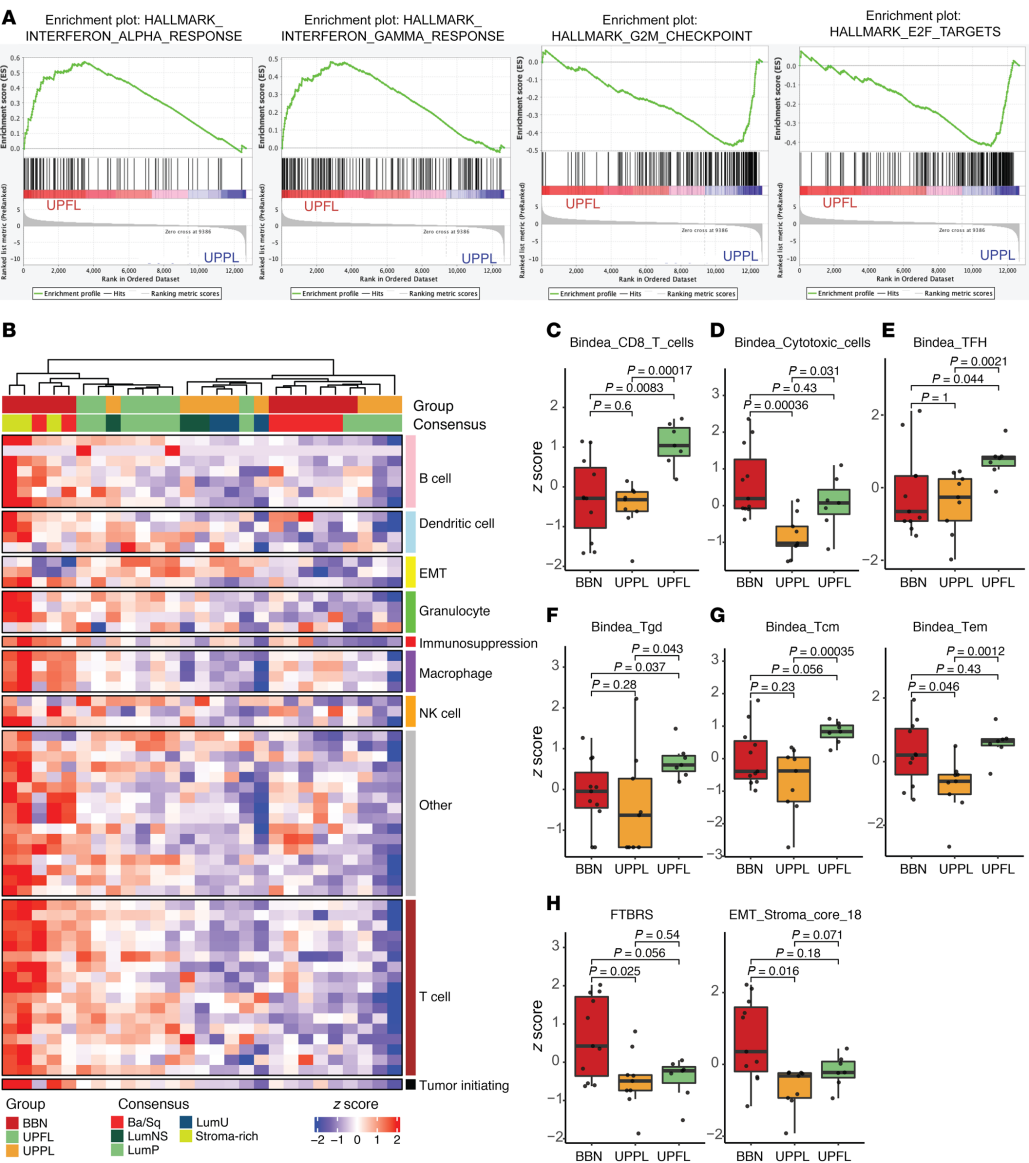
UPFL tumors have an intermediate T cell–inflamed immune contexture. (**A**) GSEA plots of the indicated gene signatures. (**B**) Heatmap of unsupervised clustering of BBN, UPPL, and UPFL primary tumors by a panel of immune gene signatures. Box-and-whisker plots of the indicated immune gene signatures of BBN, UPPL, and UPFL primary tumors: (**C**) CD8^+^ T cells, (**D**) cytotoxic cells, (**E**) T follicular helper cells (TFH), (**F**) γδ T cells (Tgd), (**G**) central memory (Tcm) and effector memory T cells (Tem), and (**H**) fibroblast TGF-β response (FTBRS) and EMT Stroma signature. All box-and-whisker plots show the IQR and midline at the median. Error bars represent Q1/Q3 ± (1.5 × IQR). Two-sided *t* tests followed by Bonferroni’s correction to account for multiple comparisons were performed, with the *P* values shown above the given comparison.

We next performed gene set enrichment analysis (GSEA), using fast GSEA (fgsea) (37), comparing *UPFL* and *UPPL* tumors and saw that the IFNA and IFNG pathways were significantly upregulated in *UPFL* tumors (relative to *UPPL* tumors) and the E2F and G_2_/M checkpoint pathways were significantly downregulated (Figure 4A and Supplemental Figure 2B). While the gene expression changes could emanate from tumor cells themselves, it is also possible that FGFR3 activation may induce a more T cell–inflamed tumor microenvironment than Pten loss in the *UPPL* model. We therefore examined the relative immune contexture of *UPFL* tumors as defined by immune gene signature expression.

By co-clustering our *UPFL* primary tumors with previously published BBN and *UPPL* tumors, we saw that BBN and *UPPL* tumors have relatively T cell–inflamed and non–T cell–inflamed immune gene signature profiles, respectively, similar to previous findings from our group (Figure 4B) (33). The *UPFL* tumors appeared to have an intermediate inflamed tumor microenvironment. This pattern is also reflected within the UROMOL data, while class 2b tumors (similar to BBN) have the highest level of immune signal, and class 1 tumors (similar to *UPFL*) have significantly higher expression of T cell signatures than class 3 (Supplemental Figure 3) (16). In a per-signature analysis, *UPFL* tumors were significantly enriched, as compared with *UPPL* tumors, for numerous subsets of T cells, including CD8^+^, central memory, and effector memory, while trending toward significance for T follicular helper cells and γδ T cells (Bindea) (Figure 4, C–G), the latter of which are important for adaptive immunity at mucosal surfaces (Figure 4G).

Finally, we examined the level of expression of fibroblast and stromal signatures (FTBRS and EMT_Stroma), which have been shown to correlate with ICI response (38, 39). While both signatures were significantly lower in *UPPL* compared with BBN tumors, *UPFL* tumors were only significantly lower that BBN tumors for the FTBRS signature (Figure 4H).

### UPFL1 and UPFL3 cell lines are sensitive to FGFR inhibition with erdafitinib.

**Figure 5:**
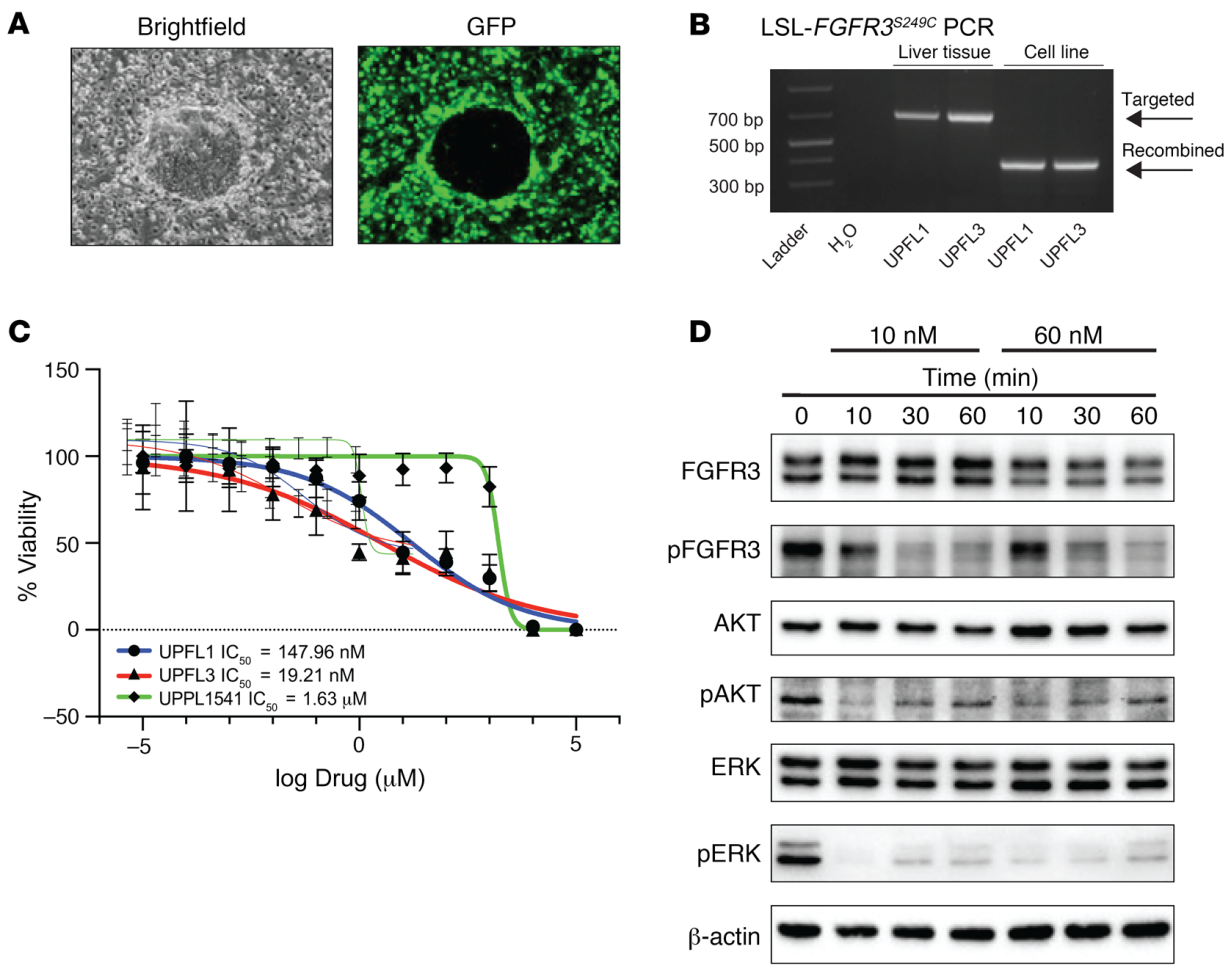
UPFL1 cell lines are sensitive to FGFR inhibition with erdafitinib. (**A**) Representative bright-field and GFP images of an epithelial island surrounded by GFP-expressing irradiated fibroblasts. (**B**) PCR demonstrating evidence of LoxP recombination of the LSL-FGFR3 S249C allele in *UPFL1* and *UPFL3* cell lines, while liver tissue shows only the targeted allele. (**C**) IC_50_ curves were generated in biologic triplicate for *UPFL1* and *UPFL3* cells’ response to erdafitinib. *UPPL1541* cells serve as a control for non–FGFR3-mutated murine bladder cancer cell line. (**D**) Immunoblots with the indicated antibodies in whole-cell extracts of *UPFL1* cells treated with erdafitinib for the indicated dose and time.

As genetically engineered mouse model (GEMM) tumors require a median of 49 weeks to form, we needed a more efficient and reproducible model to allow for the study of FGFR3-driven biology. To our knowledge, at present, there are no *FGFR3*-mutant murine cell lines that have been used to form syngeneic tumors. To this end, we generated 2 cells lines (*UPFL1* and *UPFL3* from tumors UPFL8425 and UPFL8583-1, respectively) using the conditional reprogramming of cells method described previously by Liu et al. (Figure 5A) (40). We confirmed in both cell lines the presence of recombination of the LSL cassette in the *LSL-FGFR3^S249C^* allele (Figure 5B). We next assessed the relative sensitivity to the pan-FGFR inhibitor, erdafitinib, of the *UPFL1* and *UPFL3* cell lines to our previously published *UPPL1541* cells (33). As expected, both the *UPFL1* and *UPFL3* cell lines had a low IC_50_ (15 nM and 19 nM, respectively) to erdafitinib; this was in contrast with the 1.6 μM IC_50_ for *UPPL1541* cells (Figure 5C). Moreover, erdafitinib treatment of *UPFL1* and *UPFL3* cells suppressed the MEK/ERK pathway, in keeping with the known signaling pattern seen in human cell lines (Figure 5D and Supplemental Figure 4). Therefore, the *UPFL1* and *UPFL3* cell lines are relevant models for the effects of FGFR3 inhibition in bladder tumors. Interestingly, however, the *UPFL3* cells did have rebound upregulation of p-ERK after 30 minutes of erdafitinib exposure (Supplemental Figure 4) that was not seen in *UPFL1* cells (Figure 5D); this phenomenon is still unexplained at this time.

### FGFR inhibition enhances the effect of PD-1 blockade.

**Figure 6:**
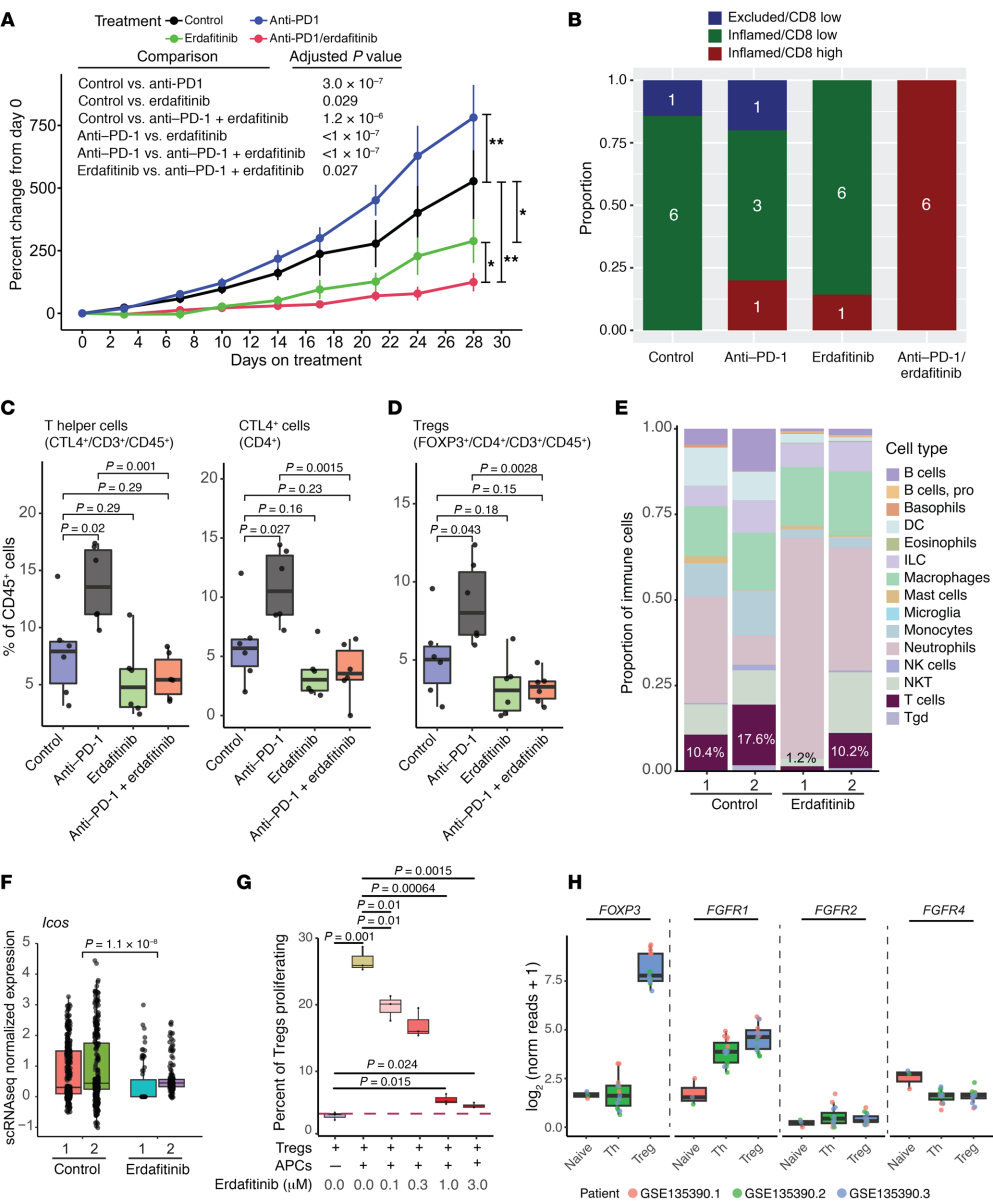
FGFR inhibition enhances the effect of PD-1 blockade. (**A**) Tumor growth curves of UPFL1 subcutaneous syngeneic tumors treated with the indicated treatments when tumor reached 150 to 300 mm^3^ in volume. Significance testing was performed by 1-way ANOVA with post hoc Tukey’s HSD. *P < 0.05, **P < 0.001. (**B**) FFPE tumors were sectioned and dual stained for CD8 and with Masson’s trichrome. Immune phenotyping was performed and the phenotype/CD8 intensity call was plotted as the proportion of each treatment group. Flow cytometry was performed on UPFL1 syngeneic tumors following 1 week of treatment. CTRL, control. (**C**) Box-and-whisker plots of percentage of cells in each treatment group of CD45^+^, CD3^+^, and CD8^+^ cytotoxic T cells after 1 week of treatment. (**D**) Box-and-whisker plot of percentage of cells in each treatment group of CD4^+^ cytotoxic T cells and CTLA-4^+^ cells after 1 week of treatment. (**E**) Cell types were assigned to scRNA-seq data from either control or erdafitinib-treated UPFL GEMM tumors using SingleR. The frequency of each immune cell type was plotted as a proportion of all immune cells. The inset percentage represents the percentage of T cells as a proportion of the total immune population. (**F**) The T cell subset of cells were plotted by the scRNA expression values for *Icos*, a marker of active/proliferative Tregs. (**G**) FoxP3^+^GFP^+^ cells were isolated from murine spleens and cocultured in the presence or absence of APCs and increasing doses of erdafitinib (*n* = 3 for each group). (**H**) Bulk RNA-seq data (GSE135390) from flow-sorted T cells (naive, Th, and Treg) were plotted for *FOXP3*, *FGFR1*, *FGFR2*, and *FGFR4*. Plots show the IQR and midline at the median. Error bars represent Q1/Q3 ± (1.5 × IQR). Two-sided *t* tests followed by Bonferroni’s correction to account for multiple comparisons were preformed, with the *P* values shown above the given comparison.

Despite having a relatively non–T cell–inflamed tumor microenvironment profile, studies have confirmed that *FGFR3*-altered tumors respond as well as *FGFR3*-WT tumors to ICI (24, 25). Nonetheless, to our knowledge, no prior work has examined how *FGFR3* alterations functionally affect the tumor microenvironment, nor how acute FGFR inhibition may cooperate with ICI in *FGFR3*-altered bladder cancer. To this end, we treated mice bearing syngeneic *UPFL1* tumors with control, erdafitinib, anti–PD-1, or the combination of erdafitinib and anti–PD-1. We found that anti–PD-1 treatment led to significantly increased tumor growth (hyperprogression) and erdafitinib treatment had significantly decreased tumor volume, relative to control treated tumors (Figure 6A), while the combination of erdafitinib and anti–PD-1 had significantly decreased tumor growth relative to all other treatment arms.

To better understand whether these changes in tumor size were related to decreased proliferation or increased cell death, we stained tumor sections from available *UPFL1* tumors with antibodies against Ki-67 (proliferation) and cleaved caspase 3 (apoptosis) (Supplemental Figure 5A). While overall we did not see significant changes in either marker, there was a trend toward decreased proliferation among all treatment groups, with the greatest effect seen in the combination anti–PD-1/erdafitinib group (Supplemental Figure 5B). Consistent with the lack of response within the single-agent anti–PD-1 group, there was no difference in cleaved caspase 3 between control and anti–PD-1; however, erdafitinib and the combination treatment both trended toward increased apoptosis (Supplemental Figure 5C).

### Erdafitinib and anti–PD-1 combination therapy promotes highly inflamed tumors.

ICI is dependent on the presence of immune cells that need to interact with tumor cells in the tumor microenvironment. To characterize the composition of the tumor microenvironment of *UPFL1* tumors following anti–PD-1 with and without FGFR inhibition, we performed both immunohistochemistry (IHC) and flow cytometry on the posttreatment *UPFL1* allografts. FFPE sections from the 4 control/treatment groups were co-stained with antibody against CD8 and Masson’s trichrome to assess IHC immune phenotype, as previously described (38) (Supplemental Figure 5D). Immune phenotype calls were then made by an expert genitourinary pathologist. While the majority of the control (6/7), anti–PD1- (3/5), erdafitinib (6/7), and erdafitinib/anti–PD-1 combination-treated (6/6) tumors were classified as inflamed, the pathologist noted variation in the CD8 staining intensity and therefore categorized inflamed tumors as CD8 high/low. Only anti–PD-1/erdafitinib–cotreated tumors (6/6) had consistently high CD8 staining, with control, anti–PD-1–, and erdafitinib-treated tumors having overwhelming low CD8 staining (Figure 6B and Supplemental Figure 5D).

### Anti–PD-1 treatment results in Treg expansion that is abrogated by concurrent FGFR inhibition.

To better ascertain the cell type composition of the treated tumors, we performed flow cytometry on *UPFL1* syngeneic tumors after 1 week of treatment with vehicle, anti–PD-1, erdafitinib, or the combination of anti–PD-1 and erdafitinib. As expected, anti–PD-1 inhibited the binding of PD-1–specific antibodies for flow cytometry on CD8^+^ and CD4^+^ T cells. Erdafitinib did not change levels of PD-1 on CD4^+^ or CD8^+^ T cells (Supplemental Figure 6A). We observed no significant changes in CD45^+^ cells, total T cells (CD3^+^), or CD8^+^ cytotoxic T cells by any treatment (Supplemental Figure 6B). Anti–PD-1–treated tumors, however, demonstrated increased numbers of CD4^+^ T cells that expressed higher levels of CTLA-4 (Figure 6C). Anti–PD-1–treated tumors also had a numerically, albeit not significantly, greater number of CD4^+^FoxP3^+^ Tregs as compared with the control tumors (Figure 6D). The increase in Tregs is similar to prior studies suggesting that the expansion of Tregs by PD-1 blockade is a mechanism of hyperprogression (41, 42). The increase in Tregs associated with anti–PD-1 treatment was abrogated in tumors that were cotreated with erdafitinib (Figure 6D). Additionally, we noticed a decrease, albeit not significant (*P* = 0.16), in the Treg population in the erdafitinib-alone group. To determine whether this was an isolated trend or could be reproduced, we reanalyzed the scRNA-seq data in which *UPFL* tumors were treated with either vehicle or erdafitinib (Figure 3). Using the cell type prediction package SingleR (43), we assigned a cell type to each of the cells present in the data set. In the 2 erdafitinib-treated tumors, T cells represented 1.2% and 10.2% of the immune cell populations, as compared with 10.4% and 17.6% for the vehicle-treated tumors (0.8% and 36% vs. 6.2% and 5.1% of total cells, respectively) (Figure 6E). We next wanted to determine whether this decrease was due to an overall reduction in the number of T cells or specific to the Treg population, which has been seen in our in vivo treatment experiment. To that end, we first examined expression of *Ptprc*, *Cd3e*, and *Cd8a*, the genes that encode CD45, CD3, and CD8, respectively. Recapitulating what we saw in the flow cytometry data, erdafitinib-treated tumors had no significant change in *Ptprc* (CD45) expression, but did have increased expression of both *Cd3e* (CD3, *P* = 0.05) and *Cd8a* (CD8, *P* = 0.007) (Supplemental Figure 6C). We next compared expression of *Icos* and *Il1r1* (CD121a), markers of suppressive and proliferative Tregs, respectively, between the vehicle- and erdafitinib-treated tumors (Figure 6F and Supplemental Figure 6D) (44–46). In both cases, erdafitinib-treated samples, as a group, had decreased expression of *Icos* (*P* = 1.1 × 10^–8^) and *Il1r1* (*P* = 0.015), suggesting that erdafitinib treatment results in less suppressive Tregs and blocks their proliferation.

In order to directly test the effect of erdafitinib on Tregs, we isolated FoxP3^+^GFP^+^ cells from the spleens of transgenic C57BL/6 mice overexpressing the diphtheria toxin receptor–eGFP (DTR-eGFP) fusion protein under control of the endogenous *Foxp3* promoter (Supplemental Figure 7) and simulated them in vitro with antigen-presenting cells (APCs) and anti-CD28 mAb; Treg proliferation was assessed as previously described (42) (Supplemental Figure 8A). At baseline, the addition of APCs and anti-CD3 to the FoxP3^+^GFP^+^ cell population induced an 8-fold increase in cell proliferation, and the addition of erdafitinib blunted the APC-induced proliferation in a dose-dependent manner, resulting in a 78% and 82% decrease at 1 μM and 3 μM concentrations, respectively (Figure 6G). Additionally, the reduction in proliferative Tregs translated to an overall increase in total percentage of cells alive at the end of the coculture experiment (Supplemental Figure 8B). Thus, these data demonstrate that FGFR inhibition reduced the proliferation of activated Tregs in a dose-dependent manner.

Prior work has suggested that FGF-1 can have a direct effect on T cells in vitro by enhancing IL-2 production and nuclear translocation of NF-κB in FGFR-bearing Jurkat T cells (36). We assessed 2 publicly available RNA expression data sets to assess *FGFR1*, *FGFR2*, *FGFR3*, and *FGFR4* expression on T cell subsets and found that while T cells, in particular Tregs, had negligible expression of *FGFR2* and *FGFR4* (*FGFR3* did not meet expression thresholds to even be included in the data set), they did express *FGFR1* (Figure 6H and Supplemental Figure 8C) (47, 48). These findings in aggregate demonstrate that the combination of erdafitinib and anti–PD-1 is superior to either single agent alone and is potentially driven by the ability of erdafitinib to abrogate anti–PD-1–induced expansion of Tregs, potentially mediated through *FGFR1*.

## Discussion

Herein, we present a mouse model of *FGFR3^S249C^*-driven UC that when combined with the *Trp53^R270H^* mutation results in high-grade NMIBC. The FGFR3-driven murine bladder tumors reflect their counterparts found in human UC. For example, RNA expression analysis of our *UPFL* tumors classify them in the consensus LumP and UROMOL class 1 MIBC and NMIBC molecular subtypes, respectively. Moreover, they have upregulated expression of luminal transcription factors such as *Gata3* as well as heightened regulon activity of luminal transcription factors PPARG and FOXA1. Our immunocompetent *UPFL* model allowed for assessment of the immunobiology underlying FGFR3-driven tumors. We found that *UPFL* tumors have an intermediate T cell–inflamed immune contexture and used our syngeneic cell line, *UPFL1*, to test the interaction between FGFR inhibition and ICI via PD-1 inhibition. *UPFL1* syngeneic tumors were sensitive to erdafitinib but notably exhibit hyperprogression with anti–PD-1 treatment, potentially due to anti–PD-1–induced Treg proliferation. In contrast, the combination of erdafitinib and anti–PD-1 worked significantly better than either alone and erdafitinib appeared to abrogate anti–PD-1–induced Treg expansion.

We saw that *FGFR3^S249^* activation promoted papillary histology as well as the development of upper tract UCs, which are known to be enriched in *FGFR3* mutations (29). Moreover, transcriptome profiling of our *UPFL* tumors demonstrated an impressive enrichment in the luminal (consensus LumP and UROMOL class 1) molecular subtypes. While this observation along with prior work from others supports the notion that FGFR3 activation promotes a luminal phenotype, a previously unresolved question in the field is whether the enrichment of *FGFR3*-altered tumors in luminal subtypes is driven by an expansion of luminal cells or whether *FGFR3* mutations drive a luminal expression pattern across all tumor cells. Our scRNA-seq profiling of control or erdafitinib-treated *UPFL* tumors demonstrates that oncogenic *FGFR3* alterations drive a luminal phenotype in all urothelial tumor cell types, suggesting that FGFR inhibition may impact a broad range of tumor cell types.

We read with interest recent work from the Allis lab suggesting that oncogenic FGFR3 activation negatively associates with luminal genes and that FGFR inhibition increases expression of luminal genes (49). Our findings appear to be in opposition to their previously published work, although there are several technical differences worth mentioning. First, it is notable that their principal component analysis (PCA) associating *FGFR3* mutations with basal gene expression is limited to TCGA LumP tumors only, rather than the entire spectrum of molecular subtypes, leaving open the possibility that their work more precisely reflects the role of FGFR3 in LumP tumors, while our studies and work from others reflects the role of FGFR3 alterations across the entire spectrum of UCs. Additionally, their work uses the small-molecule kinase inhibitor, PD-173074, to inhibit FGFR3, which in the literature has been used primarily as an FGFR1 inhibitor (50, 51). Our work, in contrast, utilizes the clinically relevant compound erdafitinib. Finally, while their studies treated immortalized human bladder cancer cell lines, our experiments were performed on tumors from autochthonous GEMMs treated in vivo. Therefore, while at face value the work by Allis and coworkers appears to be at odds with our findings, both technical differences and the spectrum of tumors examined may account for the apparent discrepancies.

A major finding from our work is the description of the *UPFL1* allograft model as a potential model for hyperprogression in response to ICI and the ability of erdafitinib when combined with ICI to abrogate this hyperprogression. Anti–PD-1–induced hyperprogression has been attributed to reversal and expansion of exhausted, PD-1–expressing Tregs (41, 42). In keeping with this notion, we found anti–PD-1–treated *UPFL1* tumors had increased numbers of Tregs. Induction of Tregs by anti–PD-1 has also seen in an FGFR2^K660N^; Trp53-mutant NSCLC GEMM, although not statistically significantly (52). In contrast, there was no evidence of Treg induction in EGFR- and KRAS-driven NSCLC GEMMs (53, 54). Therefore, the effect of anti–PD-1 on Treg abundance appears to be variable but intriguingly, the models that demonstrate anti–PD-1–induced Treg upregulation are FGFR driven. Remarkably, erdafitinib was able to completely abrogate anti–PD-1–induced Treg expansion, which was dose-dependent in an in vitro suppression assay. In mass cytometry data, we found that T cells express *Fgfr1*, but not appreciable levels of *Fgfr2*, *Fgfr3*, or *Fgfr4* and of T cell subsets, Tregs have the highest *Fgfr1* expression. We therefore propose that cell-autonomous FGFR1 inhibition on Tregs may prevent their anti–PD-1–induced reinvigoration, allowing for anti–PD-1 to work effectively. This finding is highly clinically significant, as multiple studies are currently investigating the combination of pan-FGFR inhibitors with immunotherapy in bladder cancer (i.e., ClinicalTrials.gov NCT04003610 and NCT05564416) and other cancers (i.e., ClinicalTrials.gov NCT04949191 and NCT03547037). Furthermore, there are currently large-scale efforts afoot to develop FGFR inhibitors that selectively target FGFR2 and FGFR3, which are the predominant FGFRs with activating genomic alterations. These FGFR inhibitors will have minimal FGFR1 activity. Our work suggests that at least in the context of combining FGFR inhibition with ICI, it is critical to use an agent that inhibits FGFR1 or perhaps even the development of a selective FGFR1 inhibitor. Finally, our work also puts forth the notion that combined FGFR1 and PD-1 inhibition may be effective in both FGFR-mutated and non–FGFR-mutated tumors since the effect may be driven by FGFR1 inhibition on Tregs.

In aggregate, our work reports a tractable model of *FGFR3^S249C^*-driven papillary, high-grade, NMIBC with high penetrance and that reflects the cancer biology and immunobiology of FGFR3-driven human UC. High-grade noninvasive bladder tumors are an especially clinically important area, as patients with this grade and stage are at high risk of tumor progression, which can ultimately result in the morbidity of undergoing a radical cystectomy. A tractable model of FGFR3-driven, high-grade NMIBC will be useful for therapeutic development in this disease state where extension of progression-free survival allows patients to keep their bladder as long as possible. Our scRNA-seq studies demonstrate that FGFR3 promotes luminal expression patterns across all urothelial cell types, verifying that FGFR inhibition has the ability to affect the majority of bladder tumor cells. Finally, our preclinical work has uncovered a potential role for FGFR1-mediated Treg expansion and highlights the possibility for FGFR1 inhibition as a means to prevent anti–PD-1–induced hyperprogression. This is a previously unappreciated therapeutic strategy that should be considered for drug development and explored in clinical trials.

## Methods

### Generation of ColA1-LSL-FGFR3 S249C mouse.

We generated a mouse allele with Cre-inducible expression of human *FGFR3* cDNA encoding the *S249C* hotspot mutation knocked into the *Col1a1* locus. The human *FGFR3 S249C* cDNA coding region with the Kozak sequence (GCCGCCACC) was introduced into vector pGV at the EcoRI cloning site using blunt-end cloning. Successful generation of the mutation was confirmed by sequencing. Vectors were electroporated with plasmid expressing FLP recombinase into mouse embryonic stem (ES) cells (MESC10, Mirimus) engineered with an FLP homing cassette at the *Co1A1* locus, and positive clones were identified by PCR. Positive ES clones were injected into mouse blastocysts for chimera generation. Chimeric mice were crossed with WT mice to generate mice with germline integration. The gene is expressed following Cre recombinase–mediated excision of a stop cassette flanked by LoxP sites (loxP-stop-loxP [LSL] *FGFR3* S249C/+). Both male and female mice were used in the GEMMs.

### Genotyping primers.

The AO123 (TCCAGTCTTCCTTGTGCATCC) and YL104 (GATAGGCAGCCTGCACTGGT) primers generate a 333-bp band in *LSL-FGFR3^S249C^* mice harboring a targeted allele. The AO123 and AO129 (GATGTGGGGTCCTGTCCTTT) primers generate a 572-bp band in mice with a WT *Col1A1* locus. For detecting recombination of the LSL cassette within the *LSL-FGFR3^S249C^* allele, the primers AO107 (TTCGGCTTCTGGCGTGTG) and AO106 (CGCTGCCGAAGACCAACT) were used. The targeted allele produces a 686-bp band, while after recombination, the primers produce a 376-bp PCR product.

### Mouse strains.

B6.129S4-*Trp53^tm3.1Tyj^*/J (R270H) (strain 008182), B6;DBA-Tg(Upk3a-GFP/cre/ERT2)26Amc/J (Andrew McMahon, strain 015855), *Gt(ROSA)26Sor^tm1(Luc)Kael^*/J (William Kaelin, strain 034320) (28), and *B6.129(Cg)-Foxp3tm3(Hbegf/GFP)Ayr/JFoxP3-DTR-eGFP* (strain 032050-JAX) mice were obtained from The Jackson Laboratory.

### Tamoxifen dose and administration and genotyping.

CreERT2 was activated by administration of tamoxifen (5 mg) every other day for a total of 3 times by oral gavage in 8- to 10-week-old mice. Mouse genomic DNA was isolated from a tail or toes following overnight digestion at 55°C in Nuclei Lysis Solution containing Proteinase K (Life Technologies). PCR was performed using primer pairs to distinguish WT and mutant alleles using genotyping of mouse strains as follows. The *Trp53^R270H^*, *Upk3aCreERT2*, and Rose26 LSL-Luc strains were genotyped per The Jackson Laboratory’s protocol.

### Generation of UPFL1 and UPFL3 cell lines.

Bladders were harvested from male *UFPL* mice when tumors reached a diameter of 7 mm. A portion of the tissue was taken for pathologic evaluation and the remaining tumor was dissociated and digested with collagenase and Dispase (Roche). The dissociated tumor cells were resuspended in Georgetown Media and transferred to a plastic plate as described previously (40). Cells were passaged until they propagated in DMEM independently of feeder cells. Mycoplasma testing was performed monthly while cells were in culture.

### Short tandem repeat testing.

Short tandem repeat (STR) testing was performed to establish a public database of STR profiles for our mouse cell lines. Samples were submitted to LabCorp. Eighteen mouse STR loci and 2 human STR markers (to detect human cell line contamination) were analyzed. STR profiles can be found in Supplemental Table 1.

### Cell viability assay.

Cell viability was measured using CellTiter-Glo Luminescent Cell Viability Assay (Promega) following the manufacturer’s protocols. In all cell lines, 500 cells per well were seeded into 96-well plates in triplicate, and erdafitinib (JNJ-42756493) treatment initiated after 24 hours. Cell viabilities were assessed after 96 hours. IC_50_ values were derived from the 10-dose response curves using GraphPad Prism.

### Western blot.

Whole-cell extracts were isolated using RIPA buffer supplemented with protease inhibitors and phosphatase inhibitors. The concentration of the isolated proteins was determined using Protein Assay Dye Reagent Concentrate (Bio-Rad, 5000006). Twenty micrograms of the protein were resolved in 7.5% to 10% Tris-acetate gels and electrophoretically transferred to PVDF membranes (Bio-Rad) and immunoblotted with antibodies against the following proteins: FGFR3 (C545F2) (rabbit mAb [1:1000]; Cell Signaling Technology, 4574), phospho-FGFR (Tyr653/654) (rabbit pAb [1:1000]; Cell Signaling Technology, 3471), Akt (pabbit pAb [1:1000]; Cell Signaling Technology, 9272S), phospho-Akt (Ser473) (D9E) XP (rabbit mAb [1:2000]; Cell Signaling Technology, 4060), P44/42 MAPK (Erk1/2) (rabbit pAb [1:1000]; Cell Signaling Technology, 9102S), phospho-p44/42 MAPK (Erk1/2) (Thr202/Tyr204) (E10) (mouse mAb [1:2000]; Cell Signaling Technology, 9106), β-actin (13E5) (rabbit mAb [1:1000]; Cell Signaling Technology, 5125), rabbit IgG (HRP-linked [1:1000]; Cell Signaling Technology, 7074), and mouse IgG (HRP-linked [1:1000]; Cell Signaling Technology, 7076). See complete unedited blots in the supplemental material.

### RNA/DNA extraction for RNA-seq and whole-exome sequencing.

RNA was extracted from the primary tumors and the established cell lines using an RNeasy Kit (QIAGEN) per the manufacturer’s instructions. DNA was extracted from mouse livers and cell lines using DNeasy Kit (QIAGEN) per manufacturer’s instructions.

### RNA-seq analysis.

RNA-seq libraries were prepared using a TruSeq Stranded mRNA Library Preparation Kit (Illumina, 20020595) according to the manufacturer’s protocol, and 75-bp paired-end reads were sequenced on a NextSeq 500 (Illumina). RNA reads were aligned to the mouse reference genome mm10 (ensembl) using STAR v2.5.3a (https://github.com/alexdobin/STAR) and the transcript levels were then quantified using SALMON v0.9.1 (https://combine-lab.github.io/salmon/). Gene count data were extract from SALMON output using Tximport (Bioconductor), and normalized and compared using DESeq2 (Bioconductor).

Consensus subtypes were determined by correlating the BBN, *UPPL*, and *UPFL* tumors to median expression per subtype using TCGA as the reference. The subtype was assigned based on the highest Pearson’s correlation to the given reference subtype. UROMOL class was predicted using the UROMOL predictor, as described in Lindskrog et al. (16). The basal, luminal, and Pparg signature scores were calculated based on the median expression of the genes for the indicated signature.

For immune gene signatures, *z* scores were calculated based on all genes within previously published immune gene signatures on a per-sample basis. Immune cell fractions were calculated by CIBERSORTx (https://cibersortx.stanford.edu) using the default parameters.

### scRNA-seq analysis.

CellRanger v6.1.1 (https://www.10xgenomics.com/support/software/cell-ranger) was used to demultiplex, generate FASTQ files, align reads to the mm10 reference genome, and produce a gene-cell matrix. Seurat v4.1.1 (https://satijalab.org/seurat/) was used for further quality control and data processing. Cells with feature numbers smaller than 300 or larger than the mean plus 2-fold standard deviation of feature numbers, or with over 10% mitochondria-derived feature counts were considered as low-quality cells and were removed. Doublets identified by DoubletFinder v2.0.3 (https://github.com/chris-mcginnis-ucsf/DoubletFinder) were eliminated. The remaining gene-cell matrixes were transformed by SCTransform. To regress out potential batch effects within samples, samples were integrated using IntegrateData function in the Seurat package. In order to reduce the dimensionality of the data set, PCA was performed on the integrated data matrix using the top 4,000 highly variable genes. With the ElbowPlot function in Seurat package, the top 30 PCs were used to perform downstream analysis. Cell clusters were then visualized in t-distributed stochastic neighbor embedding (tSNE) space. Cell types were assigned according to the canonical marker gene expression in each cluster, then the cell types were confirmed using per-cluster SingleR (v1.8.1) (43). Epithelial cells were subsetted for further analysis. The gene-cell matrix of epithelial cells in each sample was transformed by SCTransform separately, then they were integrated in Seurat. To reduce the dimensionality of the data set, PCA was performed using basal (*Cd44*, *Cdh3*, *Krt16*, *Krt14*, *Krt5*, *Krt6a*, *Krt6b*, and *Krt1*) and luminal genes (*Fgfr3*, *Foxa1*, *Gpx2*, *Cd24a*, *Erbb2*, *Erbb3*, *Krt19*, *Krt18*, *Krt8*, *Krt7*, *Krt20*, *Gata3*, *Pparg*, *Upk1a*, *Upk2*, *Upk3a*, *Upk3b*, and *Upk1b*). Cell clusters were visualized in tSNE space. Basal, intermediate, and luminal clusters were designated according the expression levels of basal and luminal genes in each cluster.

### Syngeneic tumor formation.

UPFL1 cells were injected subcutaneously (bilateral flank) into C57BL/6 mice, 5 × 10^6^ cells in a total of 200 μL (100 μL of PBS [Gibco, 10010049] and 100 μL of Matrigel [Corning, 354234]). Tumor volume in subcutaneous mouse allografts was measured by caliper and calculated using the formula as follows: *V* = (*L* × *W* × *W*)/2, where *L* is the tumor length and *W* is the tumor width. For efficacy studies, treatment was initiated at a tumor volume of 150 to 300 mm^3^.

### Treg proliferation assay.

CD4^+^ T cells were isolated from spleens of FoxP3^+^GFP^+^ (B6) mice and magnetically enriched for CD4^+^ T cells through magnetic isolation (EasySep Mouse T cell Isolation kit, STEMCELL Technologies) with anti-CD8a biotin (catalog 13-0081-82, clone 53–6.7, Invitrogen). FoxP3^+^GFP^+^ cells were sorted using a MACSQuant Tyto cell sorter to a purity of greater than 99%. APCs were isolated from WT B6 splenocytes and irradiated at 30 Gy. The sorted Tregs were then stained with CellTrace Violet (C34571, Invitrogen) and plated with irradiated APCs, soluble anti-CD3 (14-0031-85, eBioscience), and IL-2 (212-12, PeproTech) with or without erdafitinib in the cell culture. Cells were cultured for 3 days, stained with Zombie NIR (423105, BioLegend) and CD4-PE antibody (catalog 12-0042-82, clone RM4-5, Invitrogen), and FACS analyzed.

### Compounds and therapeutic studies.

Erdafitinib for in vitro studies was obtained from Selleck Chem (catalog S8401). For in vivo studies, anti–PD-1 and IgG2a isotype for in vivo studies were obtained from BioXcell (anti–PD-1: catalog BE0273, clone 29F.1A12 or IgG2a: catalog BE0089, clone 2A3). Anti–PD-1 or control IgG2a was administered 3 times a week (Monday, Wednesday, and Friday) via intraperitoneal injection. Erdafitinib (12.5 mg/kg) for in vivo studies was obtained from Janssen (JNJ-42756493). The in vivo vehicle consisted of 2-hydroxypropyl-β-cyclodextrin (MilliporeSigma, 332593). Both erdafitinib and vehicle control were administered by oral gavage twice a day from Monday to Friday.

### Flow cytometry.

For flow cytometry experiments on *UPFL1* allografts, treatment was initiated at tumor volume of 300 to 600 mm^3^ to allow for sufficient tumor material. Mice were treated with vehicle, erdafitinib, anti–PD-1, or a combination of erdafitinib and anti PD-1 as per *Compounds and therapeutic studies*, with the exception that erdafitinib or vehicle was given by oral gavage twice a day for 7 days.

Tumors were collected, minced into small pieces with a scalpel, and digested at 37°C for 30 minutes with a solution made of DNase I (Sigma-Aldrich, 10104159001), collagenase D (Sigma-Aldrich, 11088866001) and Hank’s balanced salt solution (Gibco, 14025-092). The digested tissue was filtered with a 70-μm cell strainer (Thermo Fisher Scientific, 22-363-548). The single-cell suspension was incubated with 1× RBC buffer (BioLegend, 420301) for 3 minutes at room temperature to lyse red blood cells. Cell pellets were washed with 1× PBS (Gibco, 10010049), spun down at 1,500 rpm for 5 minutes at 4°C, and resuspended in FACS buffer (2% FBS and 1× PBS).

The isolated tumor-infiltrating immune cells were processed for flow cytometry analysis. Live cells were determined by using Zombie Aqua fixable viability kit (BioLegend, 423101/423102). Cells were stained with surface and intracellular markers for lymphoid and myeloid population. Examples of gating strategies can be found in Supplemental Figure 6. Cells were imaged by using a BD Fortessa with FACSDiva software (v.9.0) and results were analyzed with FlowJo software (v.10.8.0).

### IHC.

Chromogenic IHC was performed on paraffin-embedded tissues that were sectioned at 5 μm. This IHC was carried out using the Leica Bond III Autostainer system. Sequential tissue sections were labeled for antigens using Ki-67 (Cell Signaling Technology, 12202S) or cleaved caspase 3 (Biocare Medical, CP229C) antibodies. Slides were dewaxed in Bond Dewax solution (Leica, AR9222) and hydrated in Bond Wash solution (Leica, AR9590). Heat-induced antigen retrieval was performed at 100°C in Bond-Epitope Retrieval solution 2 pH 9.0 (Leica, AR9640). After pretreatment, slides were incubated with anti–Ki-67 at 1:400 and anti–cleaved caspase 3 at 1:400 for 60 minutes, followed by Novolink Polymer (Leica, RE7260-CE) secondary. Antibody detection with 3,3′-diaminobenzidine (DAB) was performed using the Bond Intense R detection system (Leica, DS9263). Stained slides were dehydrated and coverslipped with Cytoseal 60 (Thermo Fisher Scientific, 23-244256). A positive control tissue was included for each run. IHC-stained slides were digitally imaged in the Aperio AT2 (Leica) using a ×40 objective.

For CD8/Masson’s trichrome, IHC was carried out using the Leica Bond III Autostainer system where tissue sections were labeled for CD8 (Cell Signaling Technology, 98941). Slides were dewaxed in Bond Dewax solution and hydrated in Bond Wash solution. Heat-induced antigen retrieval was performed at 100°C in Bond-Epitope Retrieval solution 1 pH 6.0 (Leica, AR9961). After pretreatment, slides were incubated with primary antibody diluted at 1:200 for 60 minutes followed by Novolink Polymer secondary. Antibody detection with DAB was performed using the Bond Intense R detection system. These slides were then stained using a Masson Trichrome Kit (Epredia, 87019). Afterward, slides were dehydrated and coverslipped with Cytoseal 60 (Thermo Fisher Scientific, 23-244256). A positive control tissue was included for this run. IHC-stained slides were digitally imaged in the Aperio AT2 (Leica) using a ×40 objective.

### IHC quantification.

Following staining, slides were digitized on a ScanScope AT2 slide scanner (Leica Biosystems) with a ×40 objective. The final 8-bit image per channel resolution was 0.2529 μm per pixel. Images were uploaded to eSlide Manager as JPEG-compressed Aperio SVS files and visualized with ImageScope v12.4.3 (Leica Biosystems). Images were then analyzed with the Aperio Image Processing Toolbox (Leica) using the Nuclear V9 algorithm for both Ki-67 and cleaved caspase 3 assays, as well as the Positive Pixel Count algorithm for cleaved caspase 3.

For the Nuclear V9 algorithm, which is based on the RGB color model, the average optical density (OD) values were determined for the red, green, and blue channels for both counterstain (hematoxylin) and biomarker (DAB chromogen). These input values were calculated by sampling relevant pixels from representative images. Additional input parameters included the following: Clear Area Intensity = 240 (scale of 0–255 for 8-bit image depth), Threshold lower and upper limits = 0 and 230 respectively, Smoothing = 1 μm or 4 pixels, Merging = 1.5, Trimming = Medium, Minimum Size = 10 μm^2^ or 156 pixels, Maximum Size = 1,000,000 μm^2^ or 15,635,200 pixels, Roundness = 0.1, Compactness = 0, Elongation = 0.1, Weak (1+)/Moderate (2+)/Strong (3+) Thresholds = 200/175/150.

The output data for the Nuclear V9 algorithm included number and percent positive nuclei, intensity values, and histological scores (H-scores). The H-score Excel formula was ([@[(3+) Percent Nuclei]]*3)+([@[(2+) Percent Nuclei]]*2)+([@[(1+) Percent Nuclei]]*1) to obtain scores on a scale of 0–300. These factors give extra weight to the more intensely positive nuclei.

The Positive Pixel Count algorithm is based on the Hue/Saturation/Intensity (HSI) color model. Its input parameters were as follows: Hue Value (Center) = 0.1, Hue Width = 0.1, Color Saturation Threshold = 0.05, Intensity Threshold ranges (Weak/Medium/Strong) = 175–225/125–175/0–125, respectively.

The output data for Positive Pixel Count included number, area, and intensity values for all pixels, as well as positivity (no. positive pixels/no. all pixels) and H-scores. Compression quality = 70, Compression ratio = 15–25.

### Materials availability.

Previously unpublished mouse models and cell lines generated in the course of this study are available through the request of the corresponding authors

### Statistics.

All data were collected and analyzed using RStudio 2021.09.0 build 351, R v4.1.1, and GraphPad Prism v9.5, unless otherwise noted. Analysis-specific packages have been noted within the analysis-specific methods. Statistical comparisons were performed using 2-sided *t* tests or Wilcoxon’s rank-sum test (in cases of non-normal distribution) for continuous variables. *P* values of less than 0.05 were considered significant. All box-and-whisker plots are represented by the IQR and midline at the median. Error bars represent Q1/Q3 ± (1.5 × IQR).

### Study approval.

All animals studies were approved by their respective institutional review boards: IACUC of the University of North Carolina at Chapel Hill (Chapel Hill, NC) and IACUC at NYU Langone (New York, NY).

### Data availability.

All mouse RNA-seq and scRNA-seq data have been deposited into the NCBI Gene Expression Omnibus (GEO) under accession number GSE217093 (https://www.ncbi.nlm.nih.gov/geo/query/acc.cgi?acc=GSE217093). Upper quartile–normalized RSEM gene expression data and mutation calls for TCGA-BLCA samples were downloaded from the GDC legacy archive (https://portal.gdc.cancer.gov). All additional supporting data have been provided in the supplement as the Supporting Data Values file.

## Author contributions

AO, TU, MR, JSD, KKW, and WYK conceived and planned the study. AO, TU, MR, XZ, MZ, LDP, TC, YK, DW, HH, HL, ASG, JDR, UM, AC, FS, JS, SEW, JSD, and SCG generated, analyzed, and interpreted the data. JSS generated, analyzed, and interpreted data and wrote and reviewed the manuscript. AO, TU, MR, TLR, MIM, JSD, KKW, and WYK wrote and reviewed the manuscript. AO and TU contributed equally to this work and are listed in alphabetical order.

## Supplementary Material

Supplemental data

Unedited blot and gel images

Supplemental table 1

Supporting data values

## Version 1 01/16/2024

Electronic publication
