# Supplementary material for: FGFR inhibition augments anti–PD-1 efficacy in murine FGFR3-mutant bladder cancer by abrogating immunosuppression
Source: J Clin Invest. 2024 Jan 16;134(2):e169241. doi: 10.1172/JCI169241 (PMC10786699; doi:10.1172/JCI169241)

Supplementary Figure 1

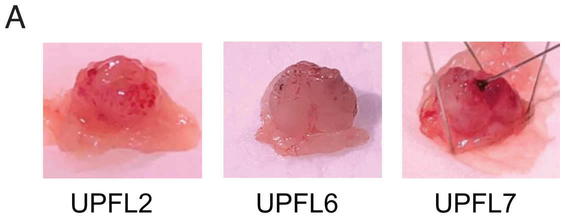

Representative images of UPFL tumors.
(A) Gross images of UPFL tumor bearing bladders.
(B-E) Photomicrographs of four (4) representative H&E stained UPFL bladder tumors. Each tumor is shown at low magnification with an inset box indicating the area of the related high magnification image.

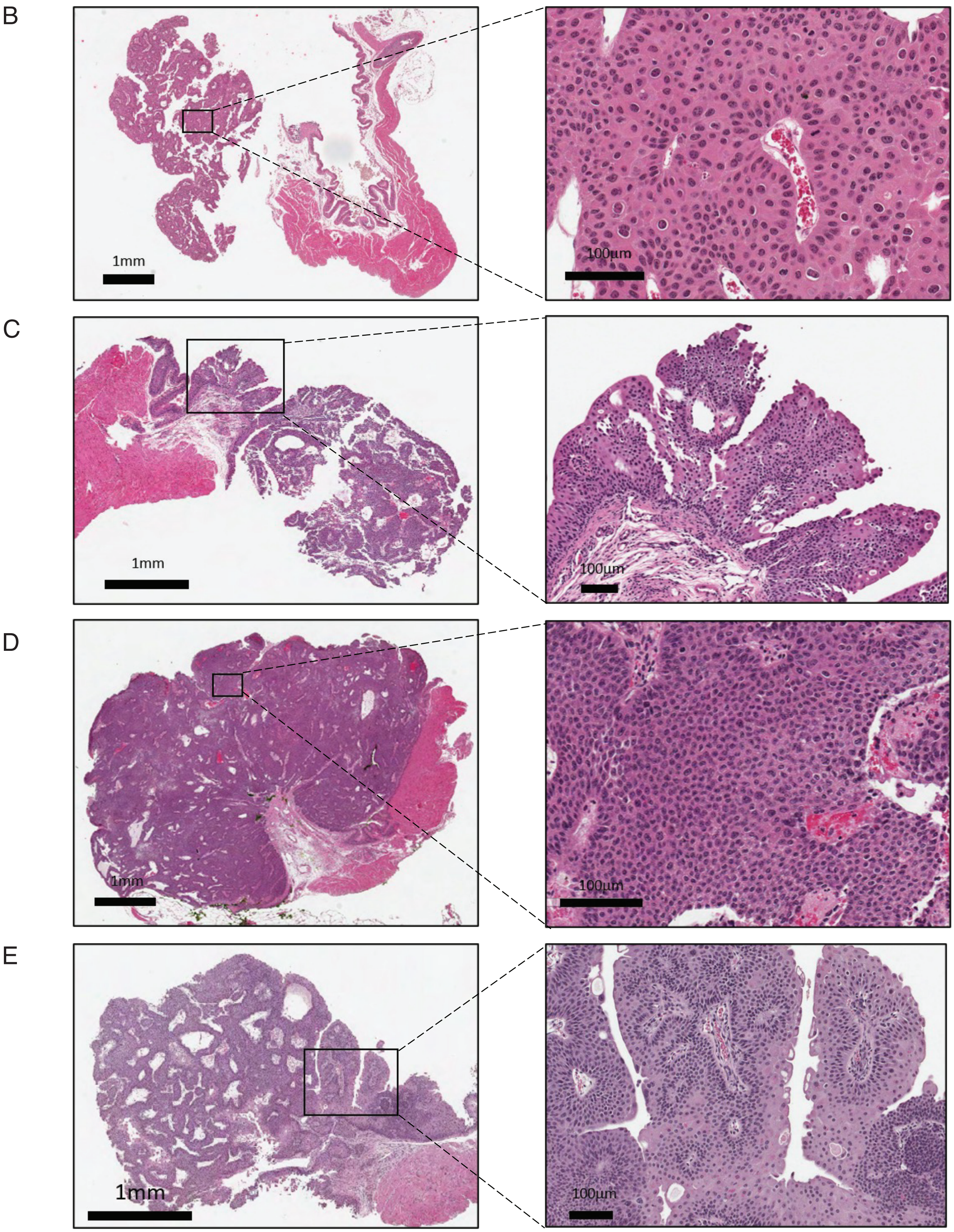

Supplementary Figure 2

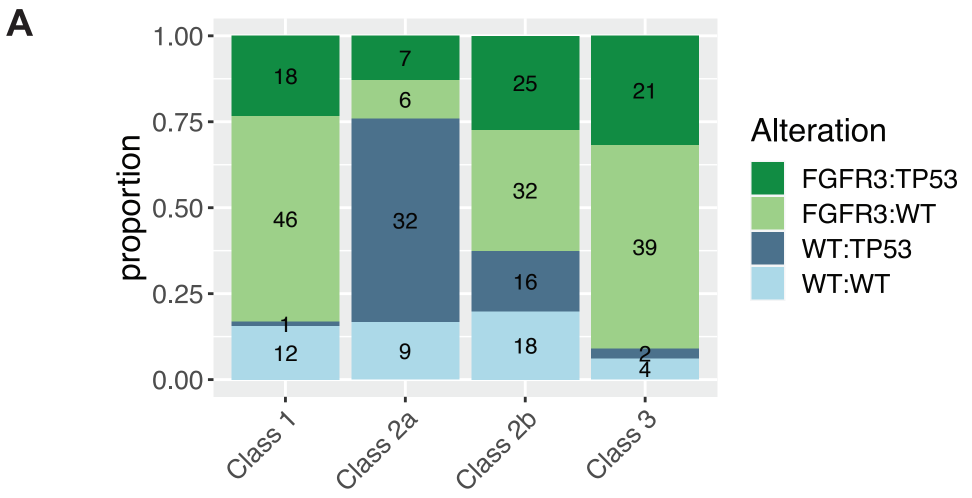

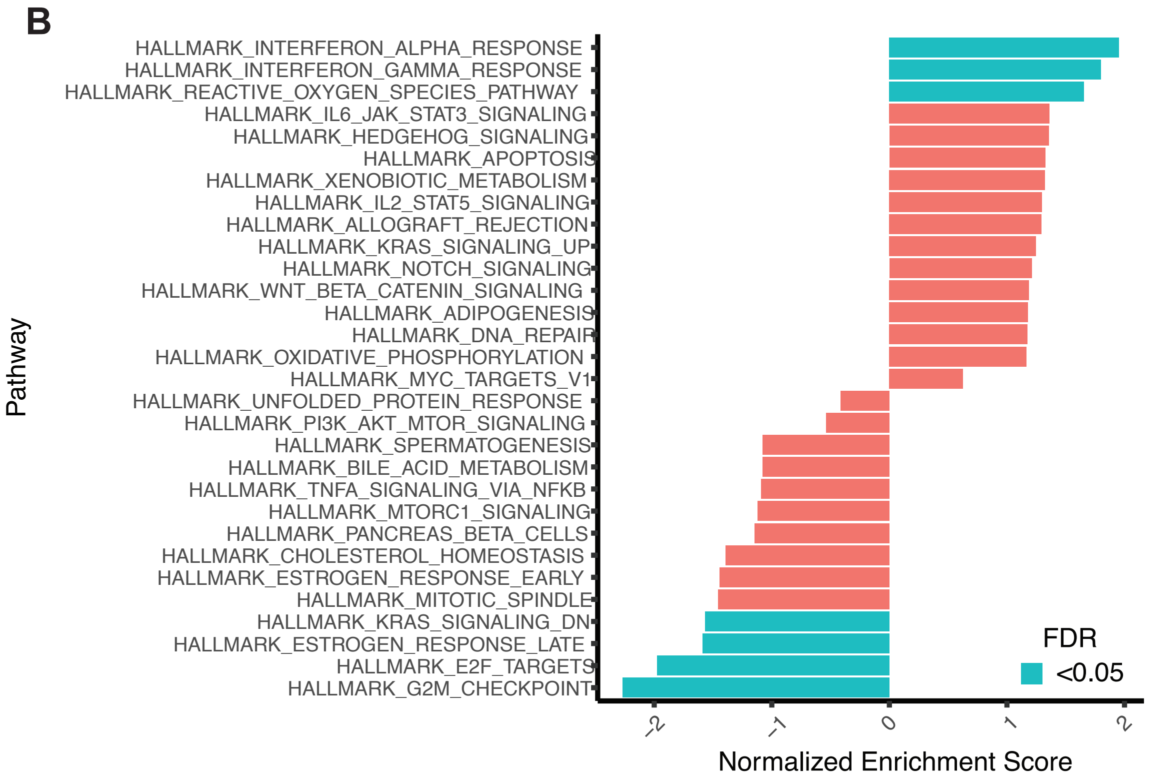

Genomic and transcriptomic features of UPFL tumors - (A) Co-occurence rates of *FGFR3* DNA calling based mutations and RNA based *TP53* Pathway alterations for the UROMOL cohort. **(B)** Gene set enrichment analysis (GSEA) for the Hallmark expression signatures were performed on the log2 - fold change between UPFL and UPPL tumors. The top ranking signatures by normalized enrichments score for UPFL and UPPL were plotted. Signature with an FDR <0.05 are indicated by teal bars.

Supplementary Figure 3

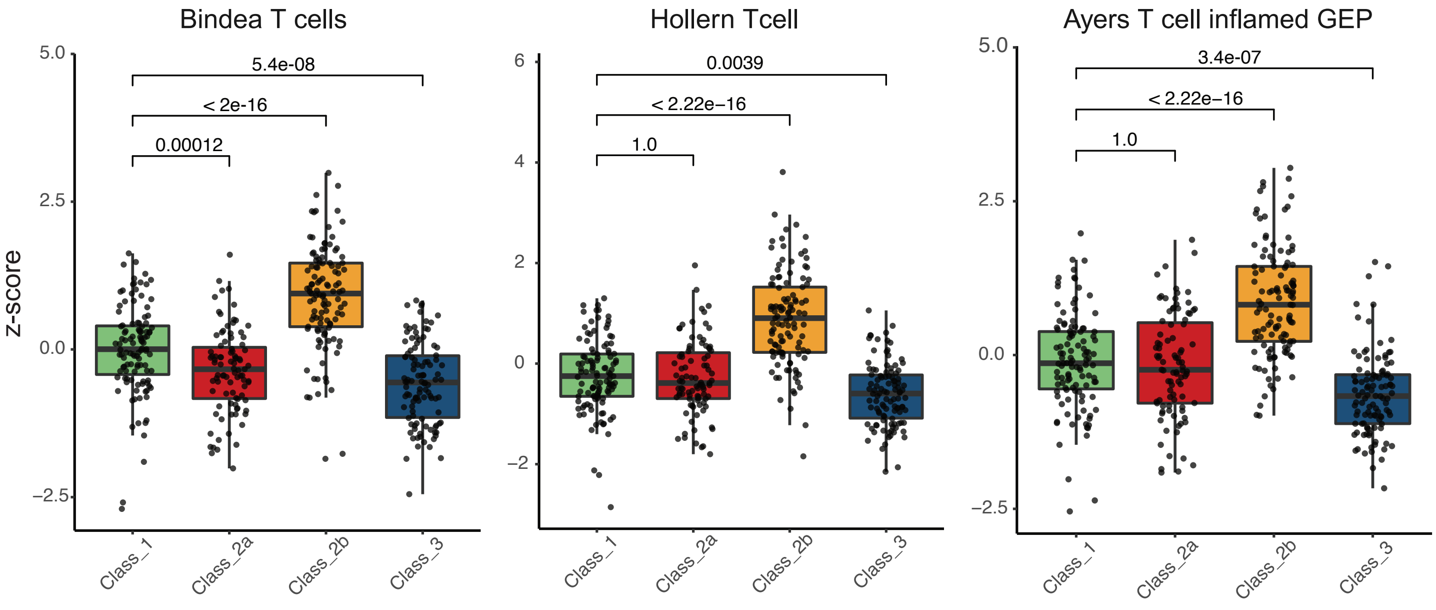

T cell signature immune gene signatures scores. Signatures representing T cells were calculated for the UROMOL cohort and plotted by NMIBC class. All boxplots are represented by the IQR and midline at the median. Error bars represent Q1/Q3 ± 1.5*IQR. Two-sided t-tests followed by Bonferroni correction, to account for multiple comparisons, were preformed with the p-values shown above the given comparison.

Supplementary Figure 4

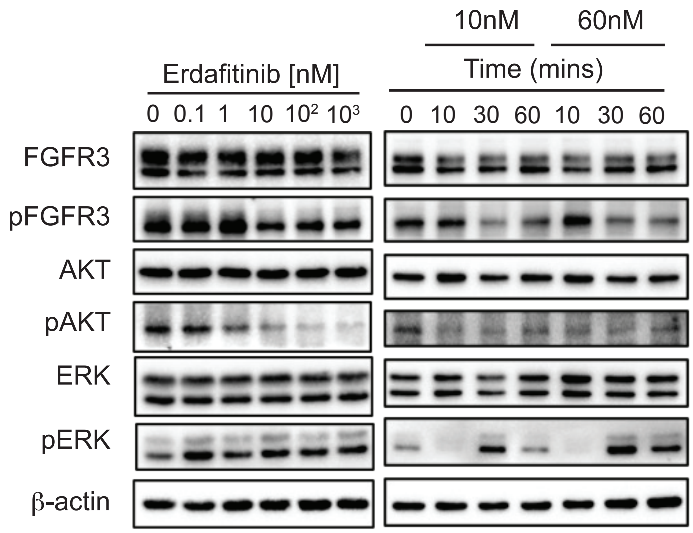

UPFL derived cell line (UPFL3) is sensitive to FGFR inhibition - Immunoblots with the indicated antibodies in whole cell extracts of UPFL3 cells treated with erdafitnib for the indicated dose and time.

Supplementary Figure 5

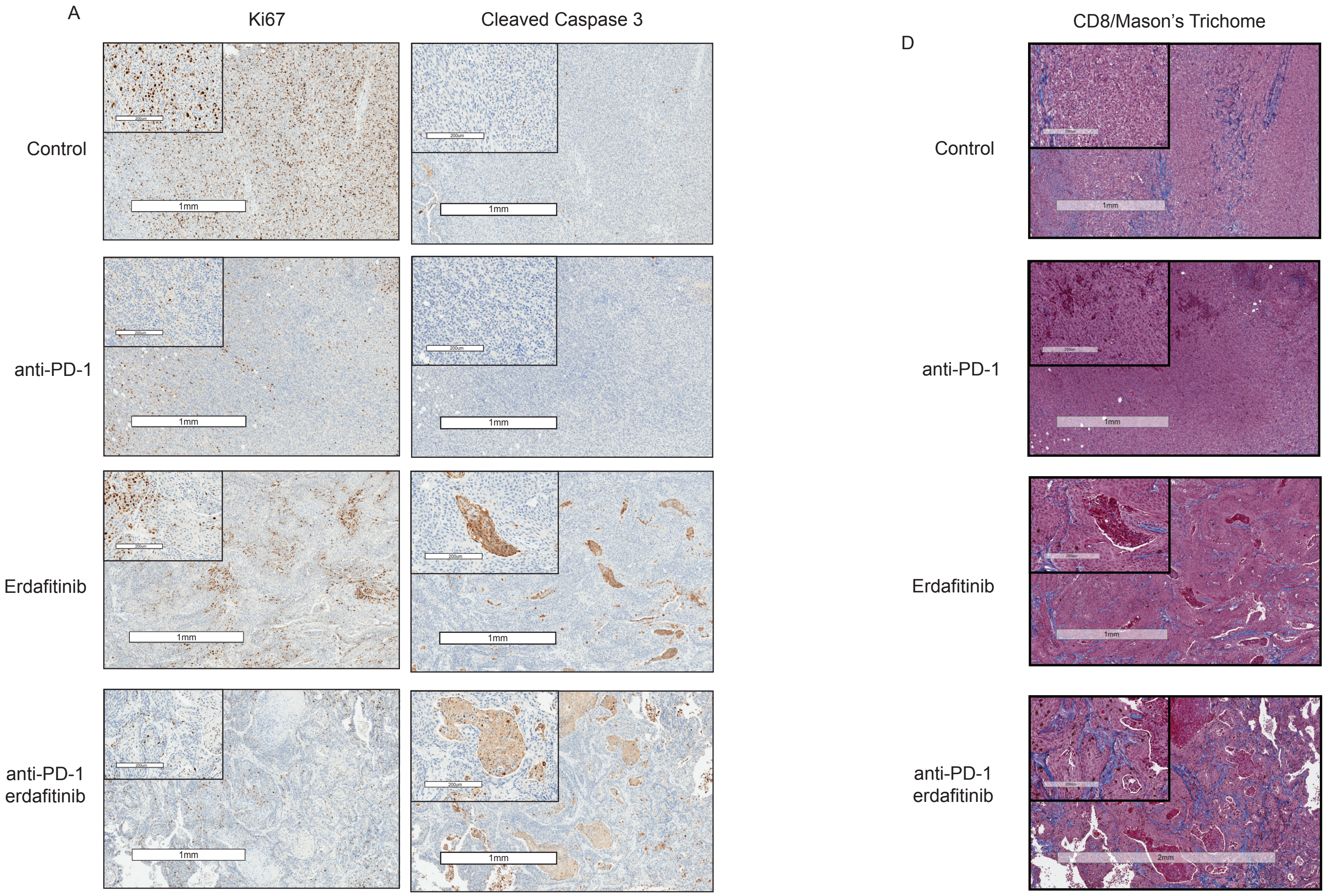

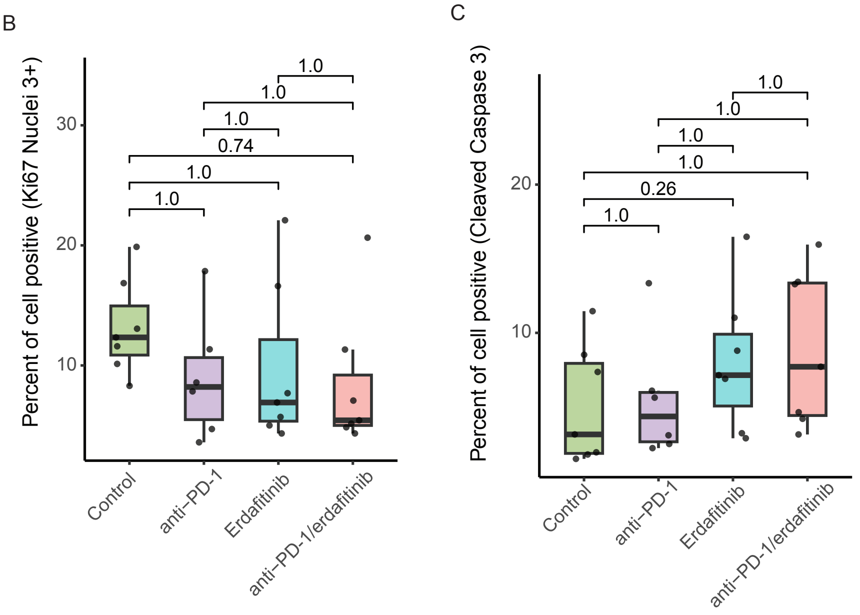

Histologic assesment of UPFL allograft tumors (A) UPFL tumors sections from mice treated with vehicle (n=7), anti-PD-1 antibody (n=5), erdafitinib (n=7), or anti-PD-1+erdafitinib (n=6) were stained for either Ki67 or Cleaved Caspase 3**. (B)** The percent of stong staining (3+) nuclei were plotted by group **(C)** along with the percent of cells positive for Cleaved Caspase 3. Boxplots are represented by the IQR and midline at the median. Error bars represent Q1/Q3 ± 1.5*IQR. Two-sided t-tests followed by Bonferroni correction, to account for multiple comparisons, were performed with the p-values shown above the given comparison. **(D)** Immune phenotype calls were made based on the location and intestity of CD8+ cells, with representive image shown above.

Supplementary Figure 6

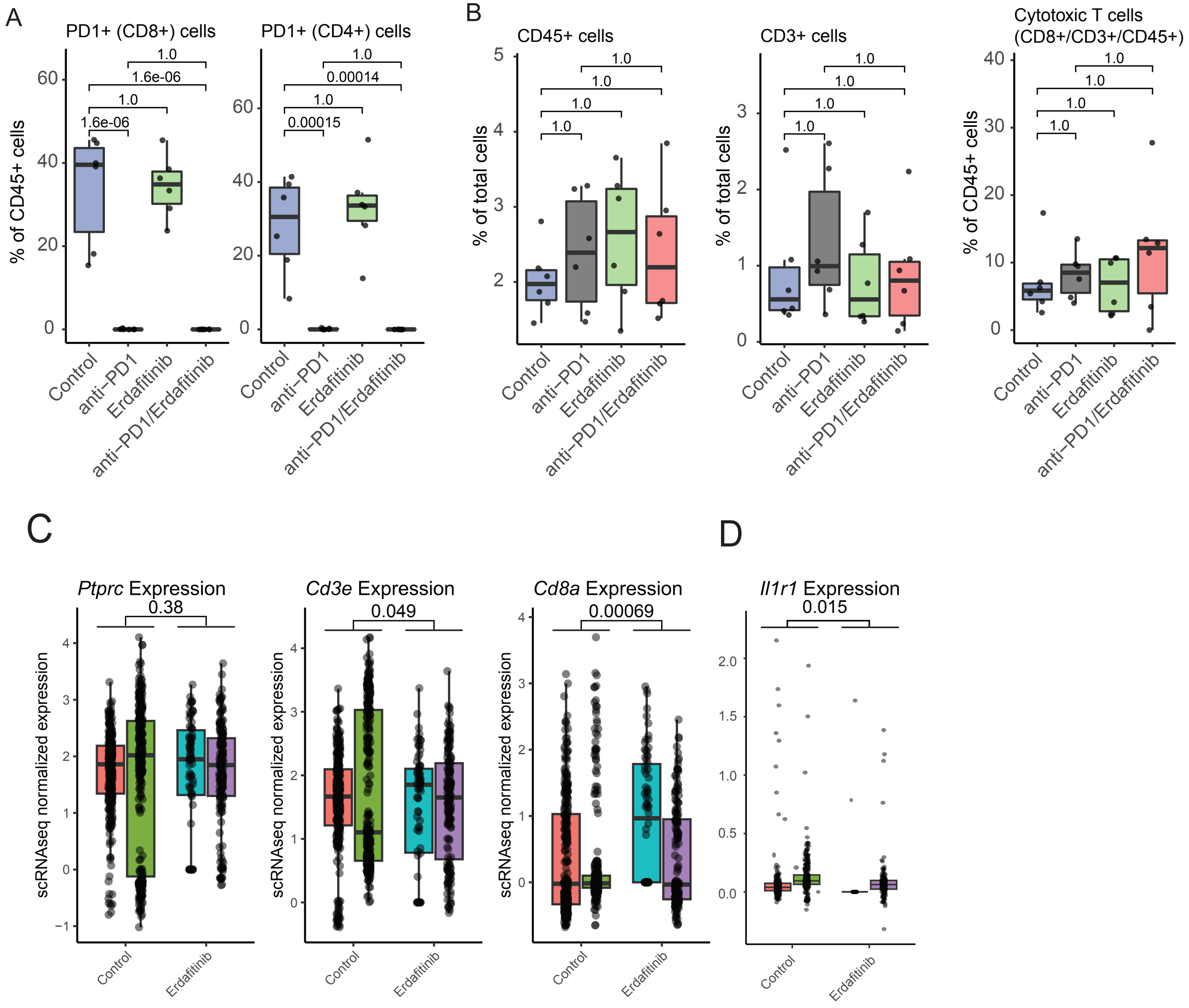

Supplementary Figure 6 (A) Box plots of percentage of CD45+ cells that are PD1+ in CD8+ (left) and CD4+ (right) for the indicated treatment group as well as **(B)** CD45+, CD3+, and CD8+ cytotoxic T cells after 1 week of treatment. **(C)** The T cell subset of cells identified by SingleR from control or erdafitinib treated tumor were plotted by the scRNA expression values for *Ptprc* (CD45), *Cd3e* (CD3), and *Cd8a* (CD8) **(D)** *Il1r1*. All boxplots are represented by the IQR and midline at the median. Error bars represent Q1/Q3 ± 1.5*IQR. Two-sided t-tests followed by Bonferroni correction, to account for multiple comparisons, were preformed with the p-values shown above the given comparison.

Supplementary Figure 7

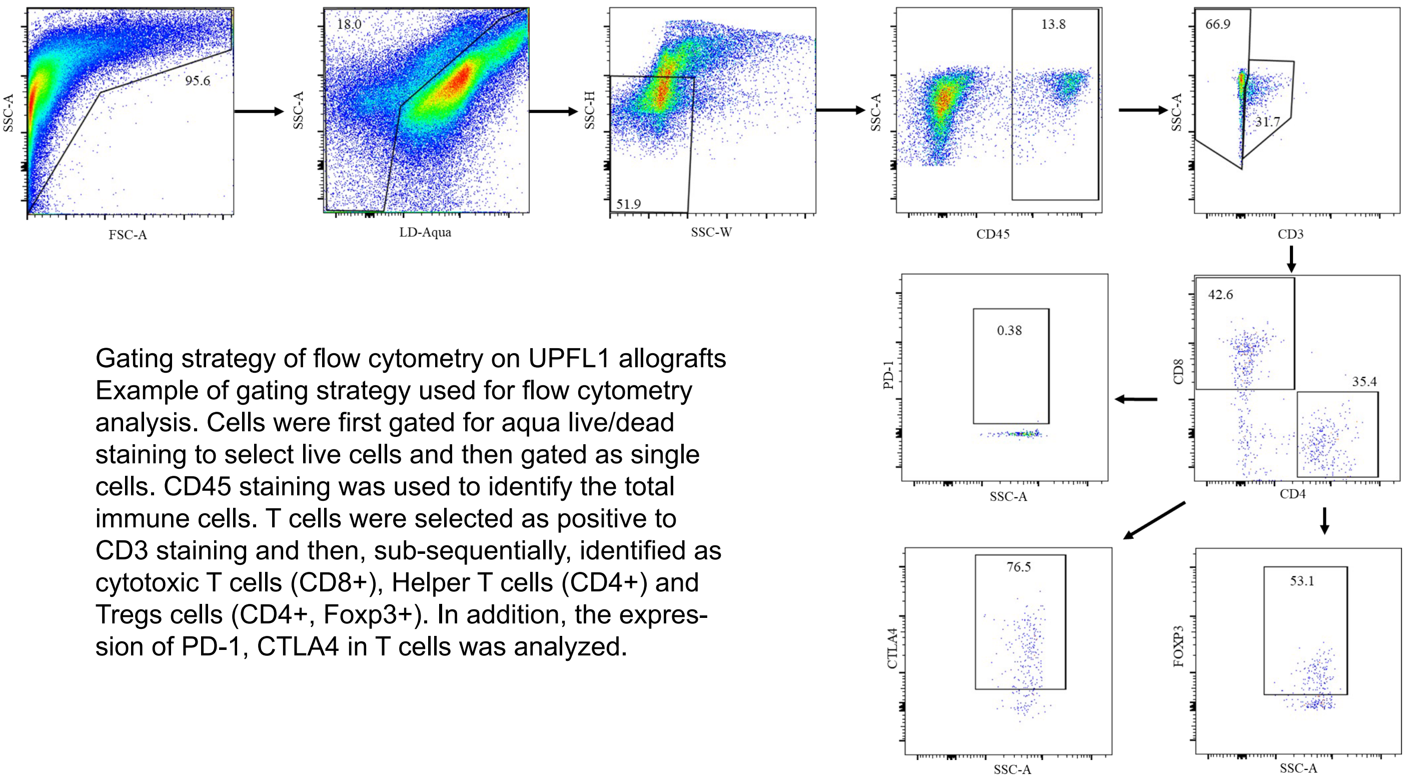

Gating strategy of flow cytometry on UPFL1 allografts
Example of gating strategy used for flow cytometry analysis. Cells were first gated for aqua live/dead staining to select live cells and then gated as single cells. CD45 staining was used to identify the total immune cells. T cells were selected as positive to CD3 staining and then, sub-sequentially, identified as cytotoxic T cells (CD8+), Helper T cells (CD4+) and Tregs cells (CD4+, Foxp3+). In addition, the expression of PD-1, CTLA4 in T cells was analyzed.

Supplementary Figure 8

A

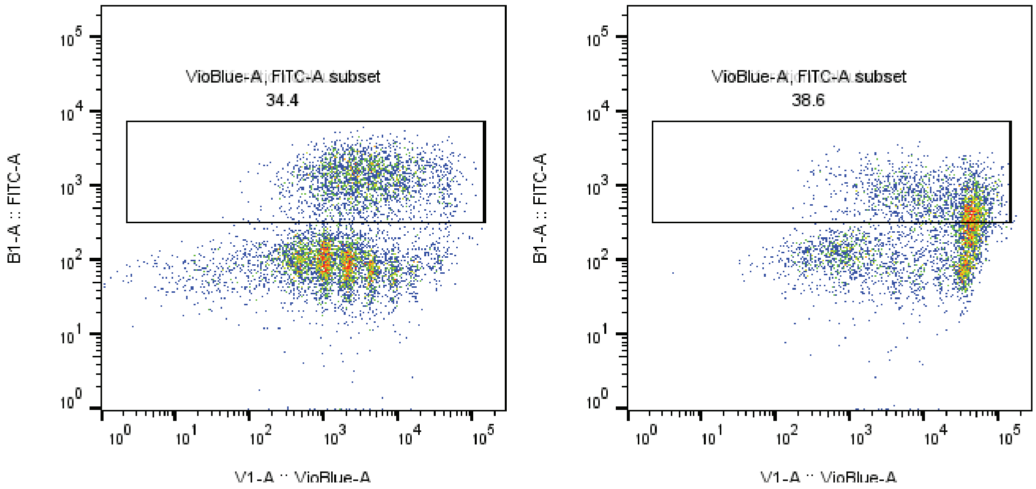

B

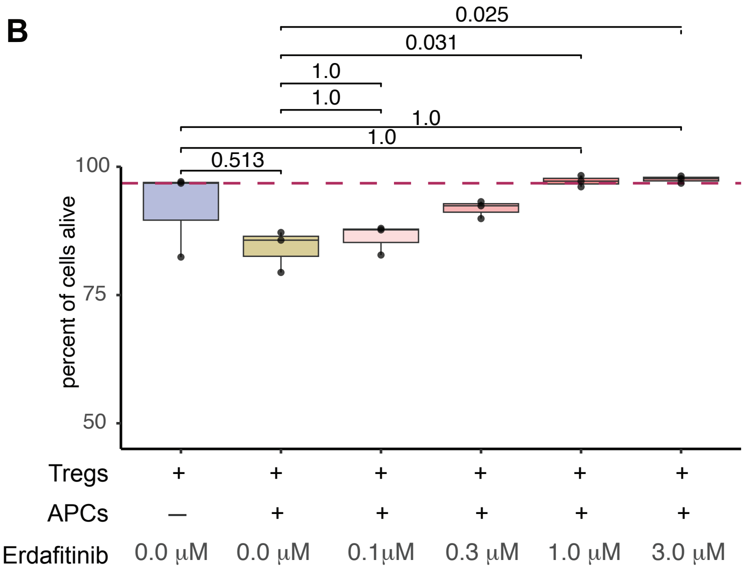

Gating stratagy and quantification for the isolation for Treg proliferation assay. (A) FACS analysis was performed on the CD4+/Foxp3-GFP+ population. Cells positive for crystal violet tracer (CVT) were identified as the non-proliferative population, where as CVT+ cells were considered proliferative. **(B)** Boxplot of the percent living cell Tregs following the 3 day proliferation assay, regardless of CVT status. (C) RNA expression of FGFR1, FGFR2, FGFR3 in T cell subsets. All boxplots are represented by the IQR and midline at the median. Error bars represent Q1/Q3 ± 1.5*IQR. Two-sided t-tests followed by Bonferroni correction, to account for multiple comparisons,were preformed with the p-values shown above the given comparison.

C

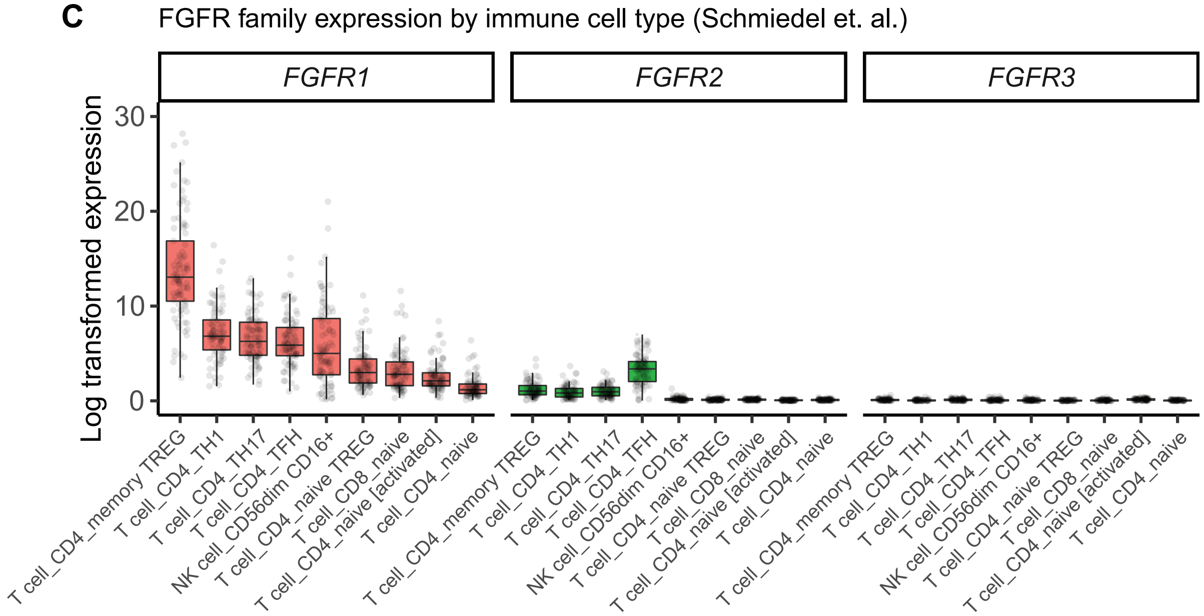

Supplement: Supplemental data [file jci-134-169241-s095.pdf]
