# Supplementary material for: FGFR inhibition augments anti–PD-1 efficacy in murine FGFR3-mutant bladder cancer by abrogating immunosuppression
Source: J Clin Invest. 2024 Jan 16;134(2):e169241. doi: 10.1172/JCI169241 (PMC10786699; doi:10.1172/JCI169241)

Uneditied western blots

Prior to immunobloting, the membrane was cut at specific molecular weights to allow for concurrent antibody incubation from the same gel.

Phosphorylated-anitibodies were probed first then stripped and subsequently re-probed for the total protein.

Figure 5d (UPFL1)

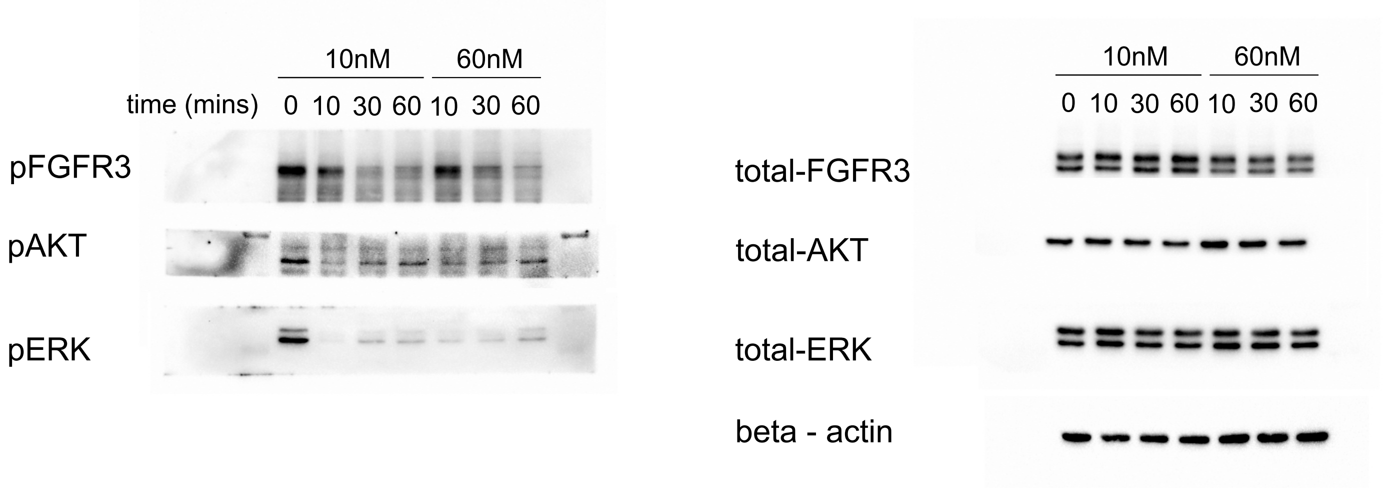

Supplementary Figure 4 (UPFL3) [Right]

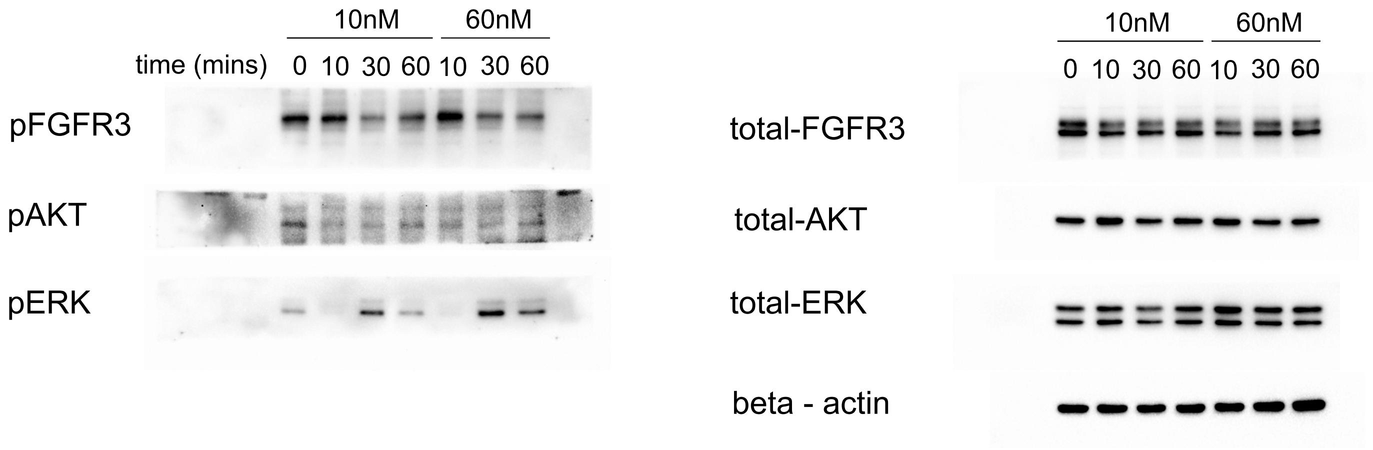

Supplementary Figure 4 (UPFL3) [Left]

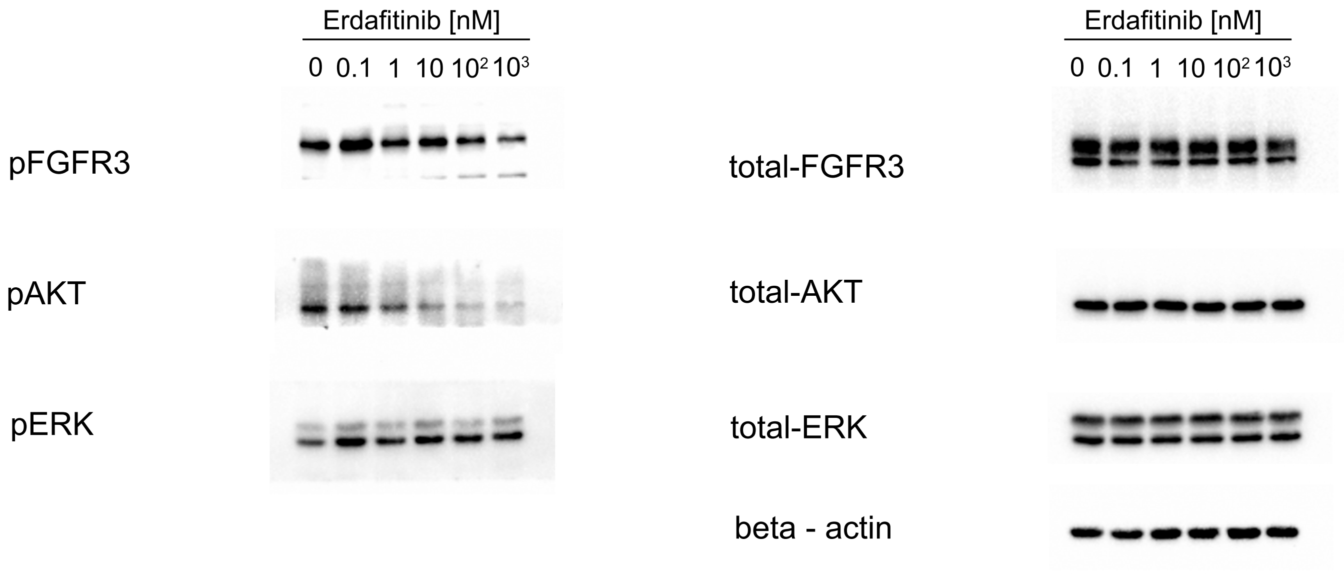

Supplement: Unedited blot and gel images [file jci-134-169241-s096.pdf]
